# Characterization of endoplasmic reticulum stress unveils ZNF703 as a promising target for colorectal cancer immunotherapy

**DOI:** 10.1186/s12967-023-04547-z

**Published:** 2023-10-11

**Authors:** Hufei Wang, Zhi Li, Yangbao Tao, Suwen Ou, Jinhua Ye, Songlin Ran, Kangjia Luo, Zilong Guan, Jun Xiang, Guoqing Yan, Yang Wang, Tianyi Ma, Shan Yu, Yanni Song, Rui Huang

**Affiliations:** 1 Department of Colorectal Cancer Surgery, the Second Affiliated Hospital of Harbin Medical University, Harbin, 150080 China; 2 Department of Obstetrics and Gynecology, the Second Affiliated Hospital of Harbin Medical University, Harbin, China; 3 Department of Breast Surgery, Harbin Medical University Cancer Hospital, Harbin, China; 4 Department of Pathology, the Second Affiliated Hospital of Harbin Medical University, Harbin, 150080 China

**Keywords:** Endoplasmic reticulum stress, Tumor immunity, Colorectal cancer, Prognosis, ICB response biomarkers

## Abstract

**Background:**

Colorectal cancer (CRC) is one of the most common malignant tumors globally, with high morbidity and mortality. Endoplasmic reticulum is a major organelle responsible for protein synthesis, processing, and transport. Endoplasmic reticulum stress (ERS) refers to the abnormal accumulation of unfolded and misfolded proteins in the endoplasmic reticulum, which are involved in tumorigenesis and cancer immunity. Nevertheless, the clinical significance of ERS remains largely unexplored in CRC.

**Methods:**

In present study, we performed an unsupervised clustering to identify two types of ERS-related subtypes [ERS clusters, and ERS-related genes (ERSGs) clusters] in multiple large-scale CRC cohorts. Through the utilization of machine learning techniques, we have successfully developed an uncomplicated yet robust gene scoring system (ERSGs signature). Furthermore, a series of analyses, including GO, KEGG, Tumor Immune Dysfunction and Exclusion (TIDE), the Consensus Molecular Subtypes (CMS), were used to explore the underlying biological differences and clinical significance between these groups. And immunohistochemical and bioinformatics analyses were performed to explore ZNF703, a gene of ERSGs scoring system.

**Results:**

We observed significant differences in prognosis and tumor immune status between the ERS clusters as well as ERSGs clusters. And the ERSGs scoring system was an independent risk factor for overall survival; and exhibited distinct tumor immune status in multicenter CRC cohorts. Besides, analyses of TNM stages, CMS groups demonstrated that patients in advanced stage and CMS4 had higher ERSGs scores. In addition, the ERSGs scores inversely correlated with positive ICB response predictors (such as, CD8A, CD274 (PD-L1), and TIS), and directly correlated with negative ICB response predictors (such as, TIDE, T cell Exclusion, COX-IS). Notably, immunohistochemical staining and bioinformatics analyses revealed that ZNF70 correlated with CD3 + and CD8 + T cells infiltration.

**Conclusion:**

Based on large-scale and multicenter transcriptomic data, our study comprehensively revealed the essential role of ERS in CRC; and constructed a novel ERSGs scoring system to predict the prognosis of patients and the efficacy of ICB treatment. Furthermore, we identified ZNF703 as a potentially promising target for ICB therapy in CRC.

**Supplementary Information:**

The online version contains supplementary material available at 10.1186/s12967-023-04547-z.

## Introduction

Colorectal cancer (CRC) is one of the most common malignant tumors globally, ranking third and second in incidence and mortality, respectively [1]. Although present therapeutic strategies such as surgery [2], radiotherapy [3], chemotherapy [4], and immunotherapy [5] have greatly improved the prognosis of patients with CRC. There is still a proportion of patients who are insensitive to these treatment regimens, become resistance at the later period of treatment, and even relapse after tumor clearance. In-depth researches on the mechanisms affecting CRC progression will help to find new intervention targets, which are expected to further improve the prognosis of CRC patients and guide clinical treatment.

Endoplasmic reticulum is a major organelle responsible for protein synthesis, processing and transport; and interacts with other organelles in the cell, such as ribosomes and Golgi complex [6], which determines the functions, fate and survival of cells. Endoplasmic reticulum stress (ERS) refers to the abnormal accumulation of unfolded and misfolded proteins in the endoplasmic reticulum under the stimulation of external factors [7]. The unfolded protein response (UPR) is then activated in an attempt to restore ER homeostasis, which is also mediated by three key sensors and their downstream signaling pathways, including *PERK*, *IRE1*, and *ATF6* [8]. Scientists have found that ERS is closely related to the occurrence and development of many diseases, such as cardiovascular diseases [9], liver diseases [10], neurodegenerative diseases [11], metabolic diseases [12]. Additionally, ERS plays an important role in the development of cancers. Studies have shown that hypoxia [13–15], nutrient deficiency [16–18], and acidosis [19, 20] in the tumor microenvironment (TME) can damage the protein folding ability of tumor cells and infiltrating immune cells, resulting in the accumulation of unfolded and misfolded protein, thus inducing ERS [7]. Appropriate UPR in tumor cells are closely associated with several oncogenic drivers. For example, activation of MYC signaling pathway also accompany activated UPR in a variety of tumors, including lymphoma, neuroblastoma, prostate cancer and breast cancer [21–24]. Whereas studies have also shown that long-term uncontrolled ERS and UPR in tumor cells induce apoptosis and inhibit tumor growth [7].

Of note, ERS in the intratumoural lymphocytes is closely related to lymphocytes mediated tumor suppression and immunotherapy efficacy. For example, neutrophils undergoing ERS overexpress low-density lipoprotein receptor 1 to acquire immunosuppressive properties through converting these cells to polymorphonuclear myeloid-derived suppressor cells [25]. The *IRE1α–XBP1* signaling has been reported to directly implicate in the polarization of macrophages, and lead to a complex immune dysregulation by modulating IL-6 and PD-L1 [26]. TME-enriched cytokines including *IL-4*, *IL-6* and *IL-10* activate *IRE1α–XBP1* signaling in macrophages facilitating cancer cell invasion [27]. The accumulation of reactive oxygen species promotes ERS and sustained *IRE1α-XBP1* activation in tumor-associated dendritic cells, thus inhibiting their ability to present antigens to intratumor T cells [28]. Furthermore, reduced expression of *ATF4* in pretreated tumor biopsy samples is associated with improved response and prolonged survival in melanoma patients treated with anti-CTLA4 [29]. Increased expression of *ATF6* in intestinal epithelial cells can induce microbial imbalance and innate immune changes, thus promoting microbial dependent colorectal tumorigenesis, and high *ATF6* expression is related to reduced disease-free survival (DFS) of CRC patients [30]. Furthermore, dehydrodiisoeugenol has been reported to inhibit the growth of CRC through ERS-induced autophagy pathways [31]. However, the clinical an immune significance of ERS in CRC is still not well understood, and more research are needed to elucidate the points.

In present study, we performed an unsupervised clustering to identify two types of ERS-related subtypes [ERS clusters, and ERS-related genes (ERSGs) clusters]. We observed significant differences in prognosis and tumor immune status between the ERS clusters as well as ERSGs clusters in multiple CRC cohorts. Through the utilization of machine learning techniques, we have successfully developed an uncomplicated yet robust gene scoring system (ERSGs signature). The ERSGs scoring system can serve as a viable substitute for the above clustering results. We also observed that the ERSGs scoring system exhibited distinct tumor immune profiles and was an independent and robust risk factor for overall survival (OS) in multicenter CRC cohorts. A series of analyses, including Tumor Immune Dysfunction and Exclusion (TIDE), the Consensus Molecular Subtypes (CMS), were then used to explore the clinical and immune significance of ERSGs signature system. Patients in advanced TNM stage and CMS4 had higher ERSGs scores. Additionally, the ERSGs scores inversely correlated with positive immune checkpoint blockade (ICB) response predictors (such as, CD8A, CD274), and directly correlated with negative ICB response predictors (such as, TIDE). Furthermore, bioinformatics analyses revealed that Zinc finger protein 703 (ZNF703), a gene of ERSGs scoring system, correlated with multiple immune indexes. The prognostic role of ZNF703 was also verified in multiple CRC cohorts. Notably, immumohistochemical staining (IHC) of our central cohort demonstrated ZNF703 was correlated with CD8 + T cell infiltration, and potentially be a promising target for tumor immunity.

## Methods

### Data source and process

GSE39582, GSE14333, GSE17536, GSE17537, and GSE72968 were microarray data of CRC cohorts on the GPL570 platform. The CEL format data of these five cohorts were downloaded from Gene Expression Omnibus (GEO) using the R package *GEOquery* [32], and processed using ReadAffy function in the *affy* package [33]. Normal samples were removed, then 1175 CRC samples were integrated as a training set (the combined GEO cohort) in this study. Background correction and standardization were carried out with robust multiarray averaging (RMA), and then the *SVA* package was used to remove batch effect among these datasets [34]. Probes corresponding to multiple genes were deleted, and the average expression was taken when multiple probes corresponded to one gene. GSE3958 with a large sample size and complete clinical data was used as the primary internal validation set. The transcriptome data (fragments per kilobase million) and clinical data of the TCGA COAD cohort were obtained from the UCSC website as an external validation set [35]. Then FPKM was converted to transcripts per kilobase million, and further log-2 transformed. The paired samples of GSE44076, GSE32323, GSE89076, and GSE113513 cohorts had been retained for exploring the gene expression level between tumor and normal tissues. The detailed information on these cohorts is summarized in Additional file 16: Table S1.

### Endoplasmic reticulum stress-related genes

A total of 878 ERS-related genes were obtained from Molecular Signatures Database, and GeneCards database as previous studies described [36, 37]. These genes were used to performed univariate Cox proportional hazards regression in the combined GEO cohort. Finally, 189 prognostic ERS-related genes were identified and used as the input genes of unsupervised clustering. These genes were summarized in Additional file 17: Table S2.

### Unsupervised clustering

A resampling unsupervised clustering method was applied for cluster the combined GEO cohort using R package *ConsensusClusterPlus* based on the input genes [38]. For this cluster algorithm, we selected the following parameters: 80% item resampling (pItem), 100% gene resampling (pFeature), a maximum evaluated k of 9 (maxK), 1000 resamplings (reps), and pam clustering algorithm (clusterAlg) upon euclidean distances (distance).

### Evaluation of the tumor microenvironment immunological characteristics

Single-sample gene set enrichment analysis (ssGSEA) implemented in R package *GSVA* [39], and Microenvironment Cell Populations-counter (MCP-counter) implemented in R package *MCPcounter* [40] algorithms were used to infer the abundance of immune cells infiltrating in the TME based on the transcriptome data. In this study, the combined GEO (1175 tumors) and TCGA COAD (471 tumors) cohorts were used for ssGSEA and MCP-counter analyses. Additionally, ssGSEA was used to calculate adaptive and innate immune scores of patients. The parameters of ssGSEA analysis were set as follows: method = 'ssgsea', KCDF = 'Gaussian'. Then, we collected 122 immunomodulators (Additional file 18: Table S3), including major histocompatibility complex (MHC), receptors, chemokines, and immunostimulants; and 20 inhibitory immune checkpoints (Additional file 19: Table S4) with therapeutic potential from previous studies [41, 42]. Cancer immunity is a reflection of the anti-cancer immune system response, and involves seven steps: release of cancer cell antigens (Step 1), cancer antigen presentation (Step 2), anticancer immune priming and activation (Step3), trafficking of immune cells to tumors (Step 4), infiltration of immune cells into tumors (Step 5), recognition of cancer cells by T cells (Step 6), and killing of cancer cells (Step 7). Successful clearance of the cancer depends on each step. The marker genes of each step were acquired from the tracking tumor immune phenotype website (TIP), and quantified using the ssGSEA algorithm to obtain an enrichment score of each step [43].

### Eleven core biological pathways

Eleven core biological pathways associated with cancers were collected from previous study [44], including *FGFR3* gene signature (FGFR3), CD8 T-effector signature (CD8 + Teff), antigen processing machinery (APM), immune checkpoint signature (ICI), *MKI67* and cell cycle genes (cell cycle), DNA replication-dependent histones (Histones), DNA damage repair genes (DDR), *TGF-β* receptor and ligand (TGFB), pan-tissue fibroblast *TGF-β* response genes (F-TBRS), angiogenesis signature (Angio), epithelial-mesenchymal transition (EMT) markers.

### Weighted correlation network analysis

Weighted correlation network analysis (WGCNA) analysis was used to identify gene modules most associated with traits [45]. The ERS clusters 1 and 2 were inputted as traits. An appropriate soft threshold β (β = 6 in this study) was calculated to meet the criteria for the scale-free network. The correlation between module genes and traits was analyzed using the Pearson method.

### Functional enrichment analyses

Gene Ontology (GO) and Kyoto Encyclopedia of Genes and Genomes (KEGG) [46] analyses were employed to explore the biological functions of the modules in WGCNA using the R package *clusterprofiler* [47]. An adjusted *P*-value of less than 0.05 was regarded as statistically significant.

### Construction and validation of ERSGs scoring system

First, we performed univariate Cox proportional hazards regression to identify the prognostic ERSGs genes using the *survival* R package. A total of 419 genes with *P*-value less than 0.01 were considered as the prognostic candidates, and inputted to least absolute shrinkage and selection operator (LASSO) regression and Random Forest algorithms [48]. After 10 cross-validations, we identified 37 genes using LASSO. And Random Forest algorithm identified 43 genes. The 12 common genes in LASSO and Random Forest algorithms were applied to multivariate Cox proportional hazards regression, which identified six genes (*CCL22*, *HOXB8*, *INHBB*, *KLK10*, *ZFP36*, and *ZNF703*) with *P*-value less than 0.05. Then, the six genes and corresponding regression coefficients in multivariate Cox proportional hazards regression were used to construct the ERSGs scoring system, as follows:

$$ERSGs\,score = \sum \limits_i \,Coefficient\,of\,\left( i \right) \times Expression\,of\,gene\,\left( i \right)$$

The regression coefficient of the gene was designated (i) in the multivariate Cox proportional hazards regression.

### Survival analysis

Only GSE39582, GSE17536, GSE17537, and GSE72968 contained comprehensive overall survival (OS) data among the combined GEO cohort (Additional file 20: Table S5). A total of 864 samples in the combined GEO cohort and 435 samples in the TCGA COAD cohort were used for survival analysis (Additional file 20: Table S5). In addition, the recurrence-free survival (RFS) data in GSE39582; disease-free survival (DFS) data in GSE17536 and GSE17537; and disease-specific survival (DSS) data in GSE17536 were summarized in Additional file 20: Table S5, which were used to validate prognostic power of the ERSGs scoring system. The survival time was converted to months format, and samples with survival time less than 1 month were excluded during survival analysis. According to the optimal cutoff value determined by the *survminer* package, the patients were divided into high and low groups. Log-rank test was employed to evaluate statistical significance. Kaplan–Meier (KM) plots were visualized using the *survminer* package.

### Identification of consensus molecular subtypes

The CMS classification is a widely used classification system currently available for CRC, and has strong prognostic implications, including CMS1 (MSI Immune), CMS2 (Canonical), CMS3 (Metabolic), and CMS4 (Mesenchymal) [49]. Among them, CMS4 characterized by prominent transforming growth factor β (TGF-β) activation, stromal invasion, and angiogenesis showed worse OS and RFS. In this study, R package *CMScaller* was used for CMS classification in the combat GEO, GSE39582, and TCGA COAD cohorts [50].

### Analysis of mutation data

The mutation data of TCGA COAD were downloaded from the TCGA website and analyzed by the “maftools” package [51]. The tumor mutation burden (TMB) was calculated using the formula: (total mutation/total covered bases) × 106.

### ICB response prediction

TIDE algorithm was employed to predict ICB response based on the gene expressions related to T cell dysfunction (Dysfunction) and T cell exclusion (Exclusion). The lower TIDE score is reportedly associated with a better immunotherapy response [52]. Furthermore, the scores of TIDE, cancer-associated fibroblasts (CAF), dysfunction, exclusion, M2 macrophages (M2), and myeloid-derived suppressor cells (MDSC) were calculated on the TIDE website. T cell inflamed score (TIS) that reflects the pre-existing anticancer immunity and positively correlated with therapeutic effect of ICB is calculated based on 18 IFN-γ-responsive genes and their corresponding weights (Additional file 21: Table S6), whose predictive power was validated in nine types of cancer, including CRC, and gastric cancer [53]. COX-2-associated inflammatory signature (COX-IS) negatively associates with ICB benefit, and validates in patient-derived tumor fragments from multiple cancer types, such as CRC, melanoma, non-small cell lung cancer, and ovarian cancer [54]. The COX-IS score was calculated using pro- and anti-tumorigenic inflammatory factors (Additional file 22: Table S7). Microsatellite instability (MSI) status is another important factor affecting ICB therapy [5], where MSI-high (MSI-H) patients are more likely benefit from ICB therapy. The MSI statuses of TCGA COAD patients were downloaded from the supplements of previous studies focusing on MSI detection [55]. There were 72 patients identified as MSI-H and 355 identified as MSI-L/MSS in TCGA COAD determined by MSI-PCR (Additional file 23: Table S8). And MSIsensor-pro [discriminative microsatellite (DMS)] scores were used as the MSI scores in this study (Additional file 23: Table S8).

### Cell culture and transfection

Human CRC cell line (HCT-116) was purchased from the American Type Culture Collection (ATCC, USA). Cells were cultured in RPMI 1640 medium (Gibco, USA) supplemented with 10% fetal bovine serum and 1% penicillin–streptomycin. The fetal bovine serum was procured from Inner Mongolia Opcel Biotechnology Co. Ltd (Hohhot, China). And cells were maintained at 37 °C in a constant-temperature incubator with a 5% CO2 atmosphere. HCT116 cells were plated at a density of 8 × 105 cells/well in six-well plates and transfected with ZNF703-overexpressing plasmids (Genechem, Shanghai, China) when they reached approximately 70% confluence. For transfection, 2.5 μg of DNA was diluted in 125 μL of Opti-MEM (Gibco, USA), followed by the addition of 4 or 5 μL of Lipo8000 (Beyotime, China). The mixture was thoroughly mixed and incubated for 10 min at room temperature. Subsequently, the transfection mixture was added to the cells in the six-well plates. After 8 h, the supernatants were replaced, and the transfected cells were collected for further experiments 48 h post-transfection.

### Protein extraction and western blotting

Total proteins were extracted from HCT116 using RIPA lysis buffer (Beyotime, China) supplemented with PMSF. Protein concentration was quantified using the BCA Protein Assay Kit (Beyotime, China). SDS polyacrylamide gels separated the proteins, which were then transferred to PVDF membranes. After blocking, primary antibodies were incubated at 4 °C overnight, followed by secondary antibodies labeled with HRP at room temperature for 2 h. Detection was performed using an ECL kit (Affinity, China). Primary antibodies included ZNF703 (Abcam # ab137054), PDL1 (Servicebio # GB11339A) and GAPDH (Abclonal # AC002).

### RNA extraction and quantitative real-time PCR analysis

Total RNAs were extracted from CRC cell lines using TRIzol™ Reagent (Invitrogen, USA). Reverse transcription was performed using PrimeScriptTM RT Master Mix (Takara, Japan) with 1 μg of total RNAs. Quantitative real-time PCR was conducted on an ABI StepOne™ Real-Time PCR System. Each 20 μL reaction was consisted of PowerUp SYBR Master Mix (Applied Biosystems), primers, template cDNA, and ddH2O. The relative mRNA abundance was determined using the 2 − ΔΔCt method, with ACTB as the internal reference gene. The primer sets used were as follows:

- Human ZNF703: Forward: 5ʹ-CTACCCGTCTCAGTTCGTGC-3ʹ,
- Reverse: 5ʹ-CAATAGGGGTCGCGGCATAA-3ʹ;
- Human ACTB:
- Forward: 5ʹ-GATTCCTATGTGGGCGACGA-3ʹ,
- Reverse: 5ʹ-AGGTCTCAAACATGATCTGGGT-3ʹ.

### Immunohistochemistry staining

For the immunohistochemistry (IHC) staining investigation, a total of 41 CRC tissues and 15 normal tissues were meticulously collected from patients who underwent surgical procedures at the Department of CRC Surgery, the Second Affiliated Hospital of Harbin Medical University (Harbin, China), from December 2022 to July 2023. This research was conducted with the ethical approval from the Ethics Committee of Harbin Medical University (Approval No. YJSKY2022-182), all patients signed informed consent. The primary antibodies used in the IHC analysis were anti-ZNF703 (abcam, #ab188031, diluted 1:200), anti-CD3 (MXB, http://maxim.com.cn, #MAB-1031, diluted 1:200), and anti-CD8 (MXB, http://maxim.com.cn, #MAB-1031, diluted 1:200). Paraffin sections were subjected to overnight incubation with the primary antibodies at 4 °C, followed by subsequent treatment with HRP-conjugated secondary antibodies at 37 °C for 60 min after PBS rinse. The tissues were counter-stained with hematoxylin and underwent DAB treatment for 2 min. The IHC results underwent independent analysis by two proficient pathologists. Regarding tumor tissues, the expression of ZNF703 was evaluated using the combined positive score (CPS) derived from IHC. CPS was calculated as the number of ZNF703–staining cells (tumor cells, lymphocytes, and macrophages) divided by the total number of viable tumor cells and multiplied by 100. The count of CD3 + and CD8 + T cells in proximity to ZNF703-staining cells was enumerated at the same view by consecutive sample sections.

### Single-cell sequencing analysis workflow

The single-cell transcriptome data for CRC were obtained from the GEO platform (project number GSE132465), encompassing 23 primary CRC samples and 10 matched normal mucosa samples [56]. Following rigorous quality control performed by the original researchers, a total of 63,689 cells were retained. Subsequently, we employed the standard process for dimensionality reduction and clustering in *Seurat* R package [57]. The *Harmony* algorithm was employed to mitigate batch effects [58]. Twenty-tow clusters were identified with the *FindClusters* function with the clustering resolution set to 0.8. We used manual annotation to determine cellular identity of each cell cluster based on marker genes from previous studies [59, 60]. Finally, cells were classified into 7 clusters: T, B, myeloid, mast, epithelial, endothelial, and mesenchymal cells.

### Statistical analysis

All analyses were performed in R 4.0.3. The difference between the two groups was tested by the Wilcox test. The log-rank test and Pearson method were used for KM survival and correlation analyses, respectively. Heat maps were visualized using the *ComplexHeatmap* package [61]. The *ggplot2* package was used to visualize boxplots, scatter plots, and sankey plots. And * represented a *P*-value less than 0.05, ** represented a *P*-value less than 0.01, *** represented a *P*-value less than 0.001, and **** represented a *P-*value less than 0.0001.

## Results

### Distinct clinical outcomes and unique biological functions evidenced in diverse ERS clusters

**Fig. 1:**
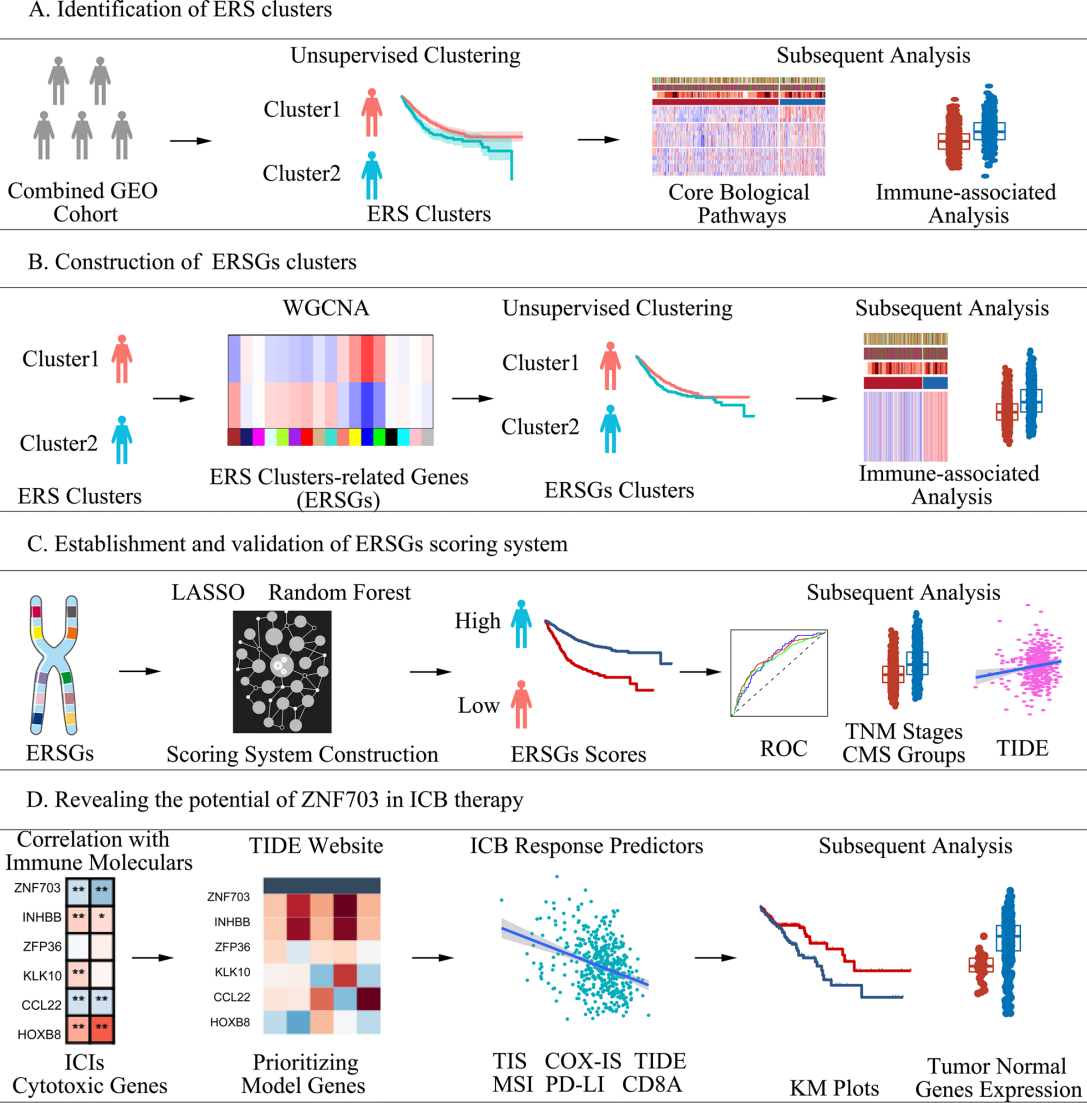
Flow diagram of this study

**Fig. 2:**
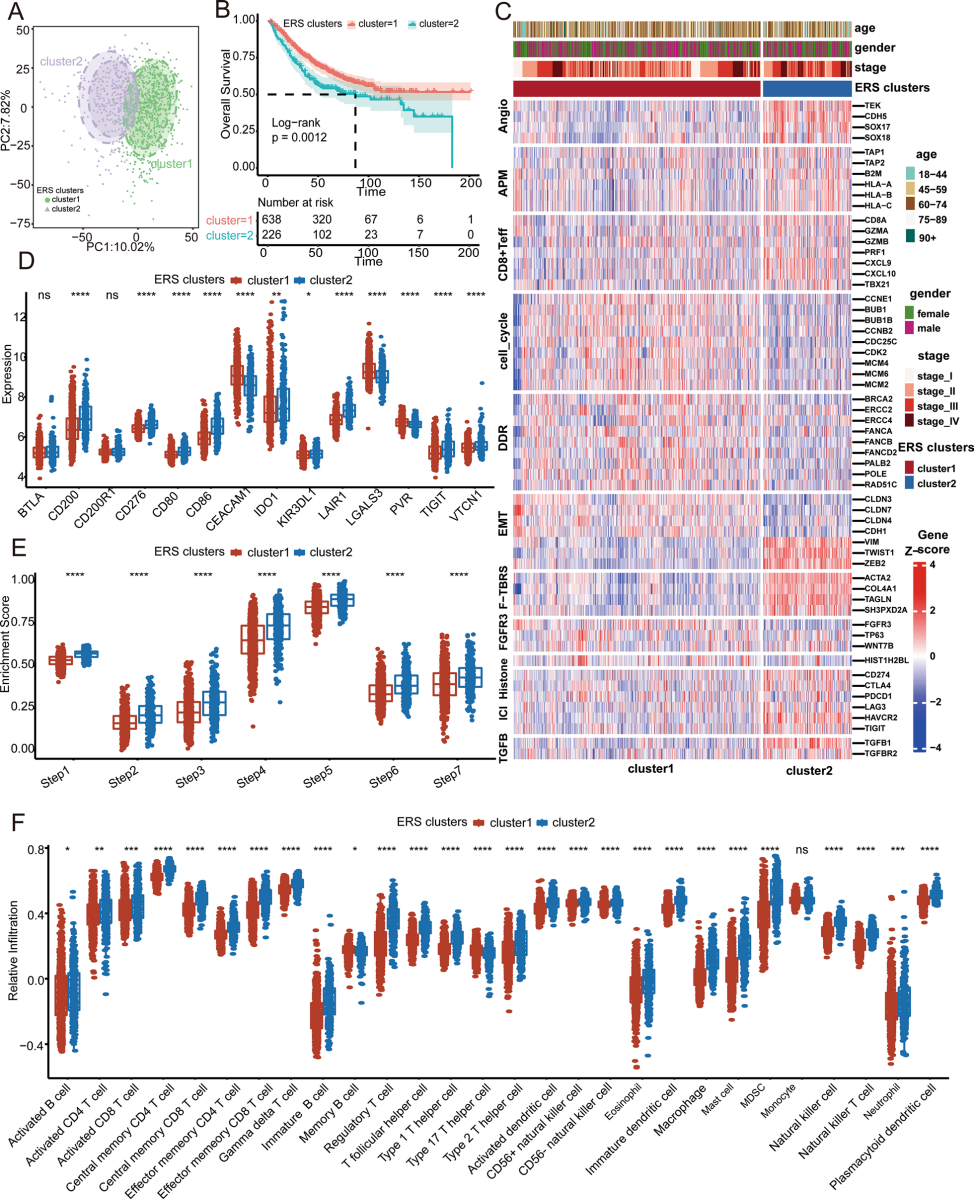
Clinical outcomes and biological functions between ERS clusters. **A** The PCA diagram shows the different gene expression patterns between ERS clusters 1 and 2. **B** KM plot shows the OS analysis of ERS clusters 1 and 2 in the combined GEO cohort. The log-rank test was used for KM survival analysis. **C** The heatmap reveals the relationships between ERS clusters and 11 critical biological pathways. Rows of the heat map represent gene expression grouped by pathway. Red and blue colors represent high and low expression, respectively. *FGFR3* gene signature (FGFR3), CD8 T-effector signature (CD8 + Teff), antigen processing machinery (APM), immune checkpoint signature (ICI), *MKI67* and cell cycle genes (cell cycle), DNA replication-dependent histones (Histones), DNA damage repair genes (DDR), *TGF-β* receptor and ligand (TGFB), pan-tissue fibroblast *TGF-β* response genes (F-TBRS), angiogenesis signature (Angio), epithelial-mesenchymal transition (EMT) markers. **D** The difference in mRNA expression of 20 inhibitory immune checkpoints between the ERS clusters. **E** The boxplot shows the differences in enrichment scores of cancer immunity cycles calculated by ssGSEA between ERS clusters. The seven steps include release of cancer cell antigens (Step 1), cancer antigen presentation (Step 2), anticancer immune priming and activation (Step3), trafficking of immune cells to tumors (Step 4), infiltration of immune cells into tumors (Step 5), recognition of cancer cells by T cells (Step 6), and killing of cancer cells (Step 7). **F** The distribution of 28 types of immune cells infiltration between ERS clusters inferred by ssGSEA analysis. And * represents a *P*-value less than 0.05, ** represents a *P*-value less than 0.01, *** represents a *P*-value less than 0.001, and **** represents a *P*-value less than 0.0001. The difference between the two groups was assessed using the Wilcox test

The flow diagram describes the construction of ERS subtypes and the ERSGs score in present study (Fig. 1). Based on 189 prognostic ERS genes, unsupervised clustering was applied to 1175 patients with CRC in the combined GEO cohort, and the clustering processes were shown in Additional file 1: Fig. S1A–D. When patients were divided into two clusters (defined as ERS clusters 1 and 2, K = 2), the clustering result was the most stable. And the PCA plot showed significant differences in gene expression profiles between the ERS clusters (Fig. 2a). Additionally, patients within ERS cluster 1 exhibited a notably superior outcome compared to ERS cluster 2 (Fig. 2b, log-rank test, *P* = 0.0012). And we performed univariate and multivariate cox regression to test whether the ERS cluster was an independent factor. Clinical factors with *P* < 0.05 in the univariate cox regression were then included in the multivariate cox regression. Results showed that the classification of ERS clusters was independent from other clinical factors, such as, tumor stage, age, gender (Additional file 24: Table S9, univariate cox *P* < 0.001, multivariate cox *P* < 0.002). Furthermore, we compared the expression levels of 11 critical biological signatures associated with tumorigenesis between ERS clusters (Fig. 2c). The findings highlighted a significant upregulation of Angio markers, and a downregulation of cell cycle-associated genes in ERS cluster 2. Additionally, immune-associated signatures, including CD8 + Teff, F-TBRS, ICI, and TGF-β, exhibited higher expression levels in ERS cluster 2 as compared to ERS cluster1. Besides the inhibitory immune checkpoints highlighted in the heatmap, most other inhibitory immune checkpoints, including *CD200*, *CD80*, *CD86*, and *IDO1*, were also elevated in ERS cluster 2 (Fig. 2d). And all the steps of cancer immunity cycles were upregulated in ERS cluster 2 (Fig. 2e). The ssGSEA analysis results showed that there were more immune cells infiltrating in the TME in ERS cluster 2, including activated B cell, activated CD4 T cell, macrophage, and neutrophils (Fig. 2f), which were validated by the MCP-counter analysis (Additional file 1: Fig. S1E). Consistently, the ssGSEA analysis unveiled a heightened activation of both the adaptive immunity and innate immunity within ERS cluster 2 (Additional file 1: Fig. S1F), indicating distinct immune statuses between ERS clusters. Subsequently, we evaluated the association between the ERS clusters and CMS classification, and found that the proportion of CMS4 patients within the ERS cluster 2 was significantly higher than that within ERS cluster 1 (Additional file 1: Fig. S1G). CMS4 has been reported to represent the mesenchymal subtype characterized by prominent TGF-β activation, stromal invasion, and angiogenesis, and displayed worse OS and RFS. To validate the robustness of ERS clusters, we also employed the same genes to cluster patients within TCGA COAD cohort. The clustering results demonstrated that the patients could still be divided into two clusters, exhibiting a remarkable concordance with the results obtained from the combined GEO cohorts (Additional file 1: Fig. S1H, Additional file 2: Fig. S2).

### Patients in ERSGs clusters 1 exhibit a better prognosis

**Fig. 3:**
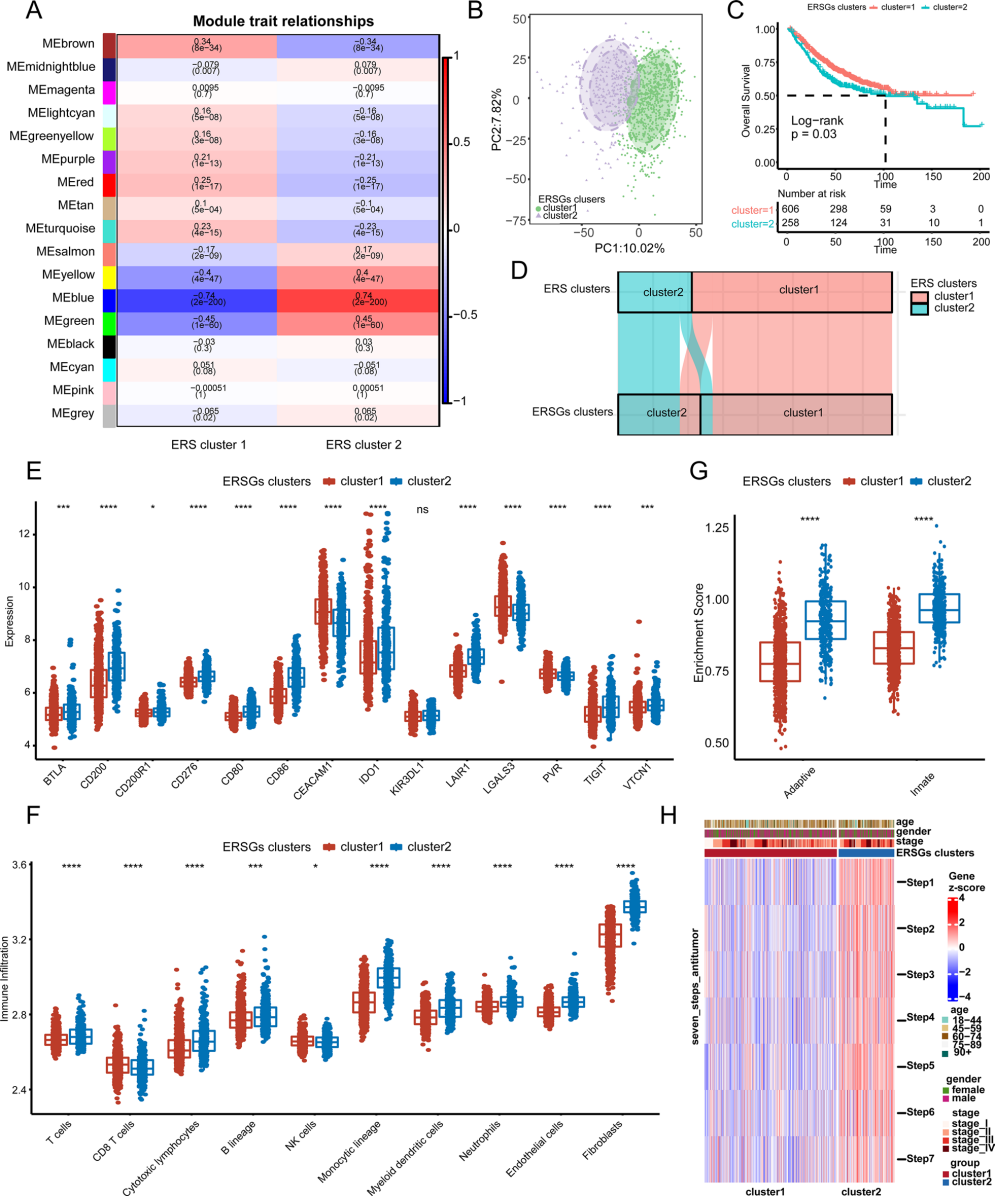
Clinical outcomes and biological functions between ERSGs clusters. **A** Correlation analysis between module eigengenes and ERS clusters. Each row contains the corresponding correlation value and *P*-value. Red and blue colors represent the positive and negative correlations, respectively. **B** The PCA diagram shows the different gene expression patterns between ERSGs clusters. **C** KM plot shows the OS analysis of ERSGs clusters in the combined GEO cohort. The log-rank test was used for KM survival analysis. **D** The sankey plot revels the relationships between the ERS clusters, and ERSGs clusters. **E** The differences in mRNA expression of 20 inhibitory immune checkpoints between the ERSGs clusters. **F** The distribution of immune cells infiltrating in the TME inferred by MCP-counter algorithm between ERSGs clusters. **G** The differences of enrichment scores of adaptive and innate immunity between ERSGs clusters inferred by ssGSEA analysis. **H** Heatmap shows the differences in cancer immunity cycles between ERSGs clusters

To identify key genes between ERS clusters 1 and 2, we performed the WGCNA analysis, and used ERS clusters 1 and 2 as the traits. The processes of WGCNA analysis were shown in Additional file 3: Fig. S3A–E. The soft threshold β = 6 meet the criteria for the scale-free network in this study. The modules-traits heatmap showed that blue (cor =  − 0.71, *P* = 2e−200), yellow (cor =  − 0.4, *P* = 4e−47), and green (cor =  − 0.46, *P* = 1e−60) modules displayed high correlations with ERS cluster 1 (Fig. 3a, Additional file 3: Fig. S3F–H). Hence, the blue, yellow, and green modules were considered as the key modules. There were respectively 605, 900, and 1089 genes in green, yellow, and blue modules (Additional file 25: Table S10), which were considered as ERS clusters-related genes (ERSGs). Then, GO and KEGG analyses were performed to explore the biological functions of genes within the key modules. When setting adjusted *P*-value of less than 0.05, GO analysis respectively identified 69, 49, and 107 terms enriched in blue, yellow, and green modules (Additional file 26: Table S11). While KEGG analysis identified 26, 26, and 53 terms enriched in in blue, yellow, and green modules (Additional file 26: Table S11). The top 15 enriched GO and KEGG terms were shown in Additional file 4: Fig. S4A, B, including *TGF-β* signaling pathway, TNF signaling pathway, IL-17 signaling pathway, cytokine-cytokine receptor interaction, and chemokine signaling pathway. Furthermore, 2594 ERSGs among blue, yellow, and green modules were again used as the input genes for unsupervised clustering in the combined GEO cohort. The clustering result was most stable when patients were divided into two groups, which were defined as ERSGs clusters 1 and 2 (Additional file 5: Fig. S5A–E). PCA plot also revealed significant differences between the ERSGs clusters (Fig. 3b). And patients in ERSGs cluster 1 exhibited better OS (Fig. 3c, log-rank test, *P* = 0.03). The sankey plot revealed that most patients in ERS cluster 1 were distributed in ERSGs cluster 1 (Fig. 3d). Consistently, the markers of Angio, CD8 + Teff, F-TBRS, ICI, and TGFB were upregulated, whereas the cell cycle markers were downregulated in ERSGs cluster 2 as compared to ERSGs cluster1 (Additional file 6: Fig. S6A). Additionally, immune-associated analyses also demonstrated that the expression of inhibitory immune checkpoints (Fig. 3e), 122 immunomodulators (Additional file 6: Fig. S6B); the abundance of immune cells infiltrating in the TME (Fig. 3f MCP-counter; Additional file 6: Fig. S6C ssGSEA); the enrichment score of adaptive and innate immunity calculated by ssGSEA (Fig. 3g), and all the steps of cancer immunity cycles (Fig. 3h) were upregulated in ERSGs cluster 2. These results demonstrated the stability of the ERS clusters, which can be validated by the key genes between the ERS clusters.

### Adverse prognosis associated with elevated ERSGs scores across various CRC cohorts

**Fig. 4:**
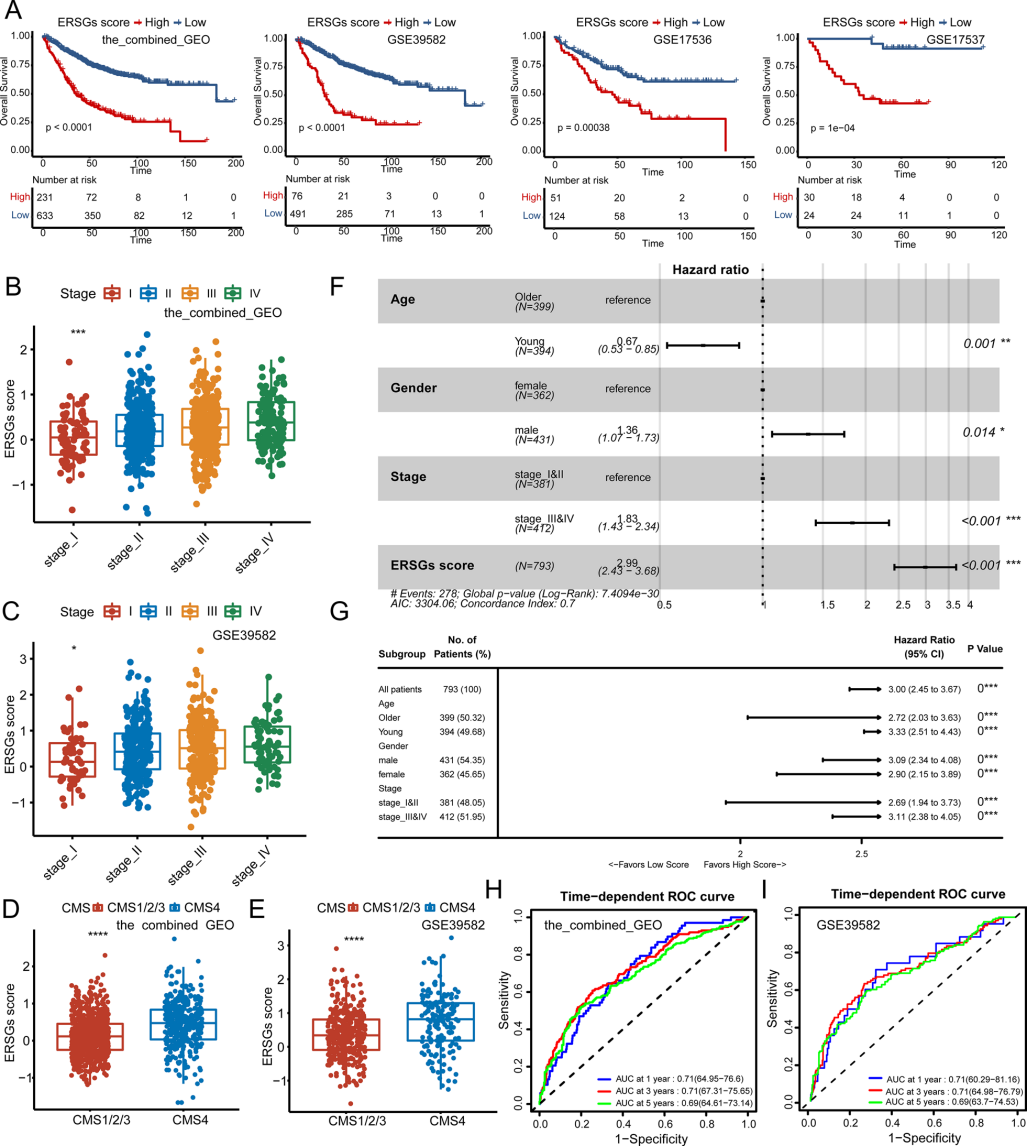
Clinical significance of ERSGs scoring system. **A** The OS analysis of ERSGs scores in the combined GEO, GSE39582, GSE17536, and GSE17537 cohorts. The log-rank test was used for KM survival analysis. **B**, **C** The distribution of ERSGs scores in different TNM stages. The statistic differences are assessed by the Kruskal test. **D**, **E** The distribution of ERSGs scores in different CMS groups. The statistic differences are assessed by the Wilcox test. **F** Multivariable Cox regression analysis of OS in the combined GEO cohort. **G** Subgroup survival analysis of ERSGs scoring system in different age, gender, and TNM stages in the combined GEO cohort. **H**, **I** Time-dependent ROC analysis for predicting OS at 1, 3, and 5 years in the combined GEO and GSE39582 cohorts, respectively

Scoring systems are simple and effective models widely applied in clinical practice [62–64]. To further facilitate the application of ERS subtypes in CRC, we aimed to establish a scoring system based on the 2594 ERSGs identified by WGCNA analysis, named ERS clusters-associated genes (ERSGs) scoring system. First, 2594 ERSGs were used for univariate Cox regression analysis in the combined GEO cohort. With a *P*-value less than 0.01, 419 prognostic genes were obtained (Additional file 27: Table S12). Furthermore, Lasso regression and Random Forest algorithms were used to select critical genes for constructing the scoring system. The process of Lasso regression was showed in Additional file 7: Fig. S7A, B. A total of 37 and 43 genes were identified by the Lasso regression and Random Forest algorithms, respectively (Additional file 27: Table S12). And the 12 common genes were subjected to multivariate Cox regression, which finally identified a set of six genes (*CCL22*, *HOXB8*, *INHBB*, *KLK10*, *ZFP36*, and *ZNF703*). Next, the ERSGs score was calculated according to the expression of the six genes weighted by their regression coefficients in multivariate Cox regression model (Additional file 28: Table S13). All patients were assigned into high and low ERSGs score groups based on the optimal cut-off value determined by the *survminer* package. Excitingly, OS analysis demonstrated that patients with high ERSGs scores exhibited worse prognosis than patients with low ERSGs scores (Fig. 4a, log-rank test, *P* < 0.0001), which were also validated in internal validation cohorts, including GSE39582, GSE17536, and GSE17537 (Fig. 4a, log-rank test, *P* < 0.0001). In addition, the RFS, DFS and DSS in the low ERSGs score group were superior to those of the high ERSGs score group (Additional file 7: Fig. S7C, GSE39582 RFS, GSE17537 DFS, GSE17536 DFS, GSE17536 DSS). GSE39582 was a large size cohort with complete clinical information that was used as the primary internal validation set in the subsequent analysis. And patients in advanced stages (stage III&IV) exhibited significantly higher ERSGs scores in the combined GEO, and GSE39582 cohorts (Fig. 4b, c). Furthermore, the CMS classification were inferred in the combined GEO and GSE39582 cohorts using the *CMScaller* package (Additional file 29: Table S14). We observed that patients in CMS4 had higher ERSGs scores than the other subtypes (Fig. 4d, e), which were in accordance with the indications of the ERSGs scores. Furthermore, multivariate Cox regression demonstrated that ERSGs scoring system was still a robust predictor for OS after adjusting for common clinicopathological parameters, such as age, gender, and stage in the combined GEO and GSE39582 cohort (Fig. 4f, Additional file 7: Fig. S7D). And subgroup analysis showed that ERSGs scoring system predicted the worse OS within subgroups of age, gender, and stage (Fig. 4g, Additional file 7: Fig. S7E all *P*-value < 0.001). Next, to evaluate the prognostic power of the ERSGs scoring system, we performed ROC analysis and observed that 1-, 3-, 5-year AUCs of 0.71, 0.71, and 0.69 in the combined GEO and GSE39582 cohorts (Fig. 4h, i).

### Validating the performance of ERSGs scoring system in the TCGA COAD cohort

**Fig. 5:**
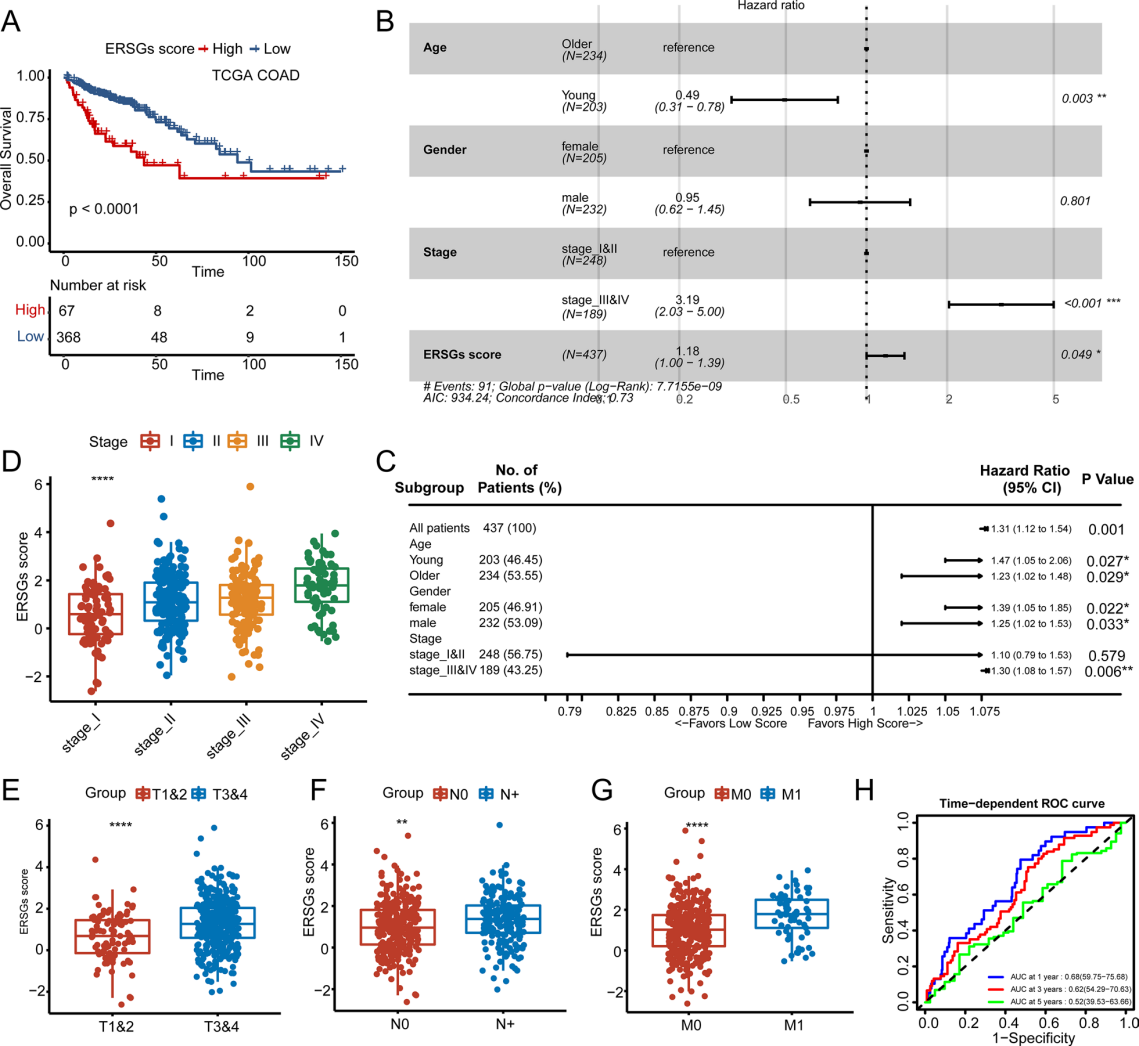
Validation of the clinical significance of ERSGs scoring system in TCGA COAD cohort. **A** The OS analysis of ERSGs scoring system in the TCGA COAD cohort. The log-rank test was used for KM survival analysis. **B** Multivariable Cox regression analysis of OS in the TCGA COAD cohort. **C** Subgroup survival analysis of ERSGs scoring system in different age, gender, and TNM stages in the TCGA COAD cohort. **D**–**G** The distribution of ERSGs scores in different TNM, T, N, and M stages in TCGA COAD cohort. The statistic differences in more than two groups are assessed by the Kruskal test. The difference between the two groups was assessed using the Wilcox test. **H** Time-dependent ROC analysis for predicting OS at 1, 3, and 5 years in the TCGA COAD cohort

To further verify the performance of our ERSGs scoring system, the TCGA COAD cohort was used as the external validation set. Consistently, patients with high ERSGs scores exhibited dramatically worse OS (Fig. 5a, log-rank test, *P* < 0.0001). Multivariate Cox regression demonstrated that ERSGs scoring system was still a robust, and independent predictor for OS after adjusting for common clinicopathological parameters, such as age, gender, and stage (Fig. 5b). And subgroup analysis showed that ERSGs scoring system predicted the worse OS within subgroups of young, older, male, female, and stage III&IV, except stage I&II (Fig. 5c, *P*-value = 0.027, 0.029, 0.022, 0.033, and 0.006). Although, no significant difference was observed between CMS4 and the other subtypes in the ERSGs scores (Additional file 8: Fig. S8A), patients in advanced stages (stage III&IV) exhibited significantly higher ERSGs scores (Fig. 5d). In addition, we observed that the ERSGs scores of patients were significantly higher in T3&4, N+, and M0 stages than T1&2, N0, and M1, respectively (Fig. 5e–g). The ROC analysis showed that 1-, 3-, 5-year AUCs of 0.68, 0.62, and 0.52 in the TGCA COAD cohorts (Fig. 5h).

### Potential favorable response to ICB in patients with low ERSGs scores

**Fig. 6:**
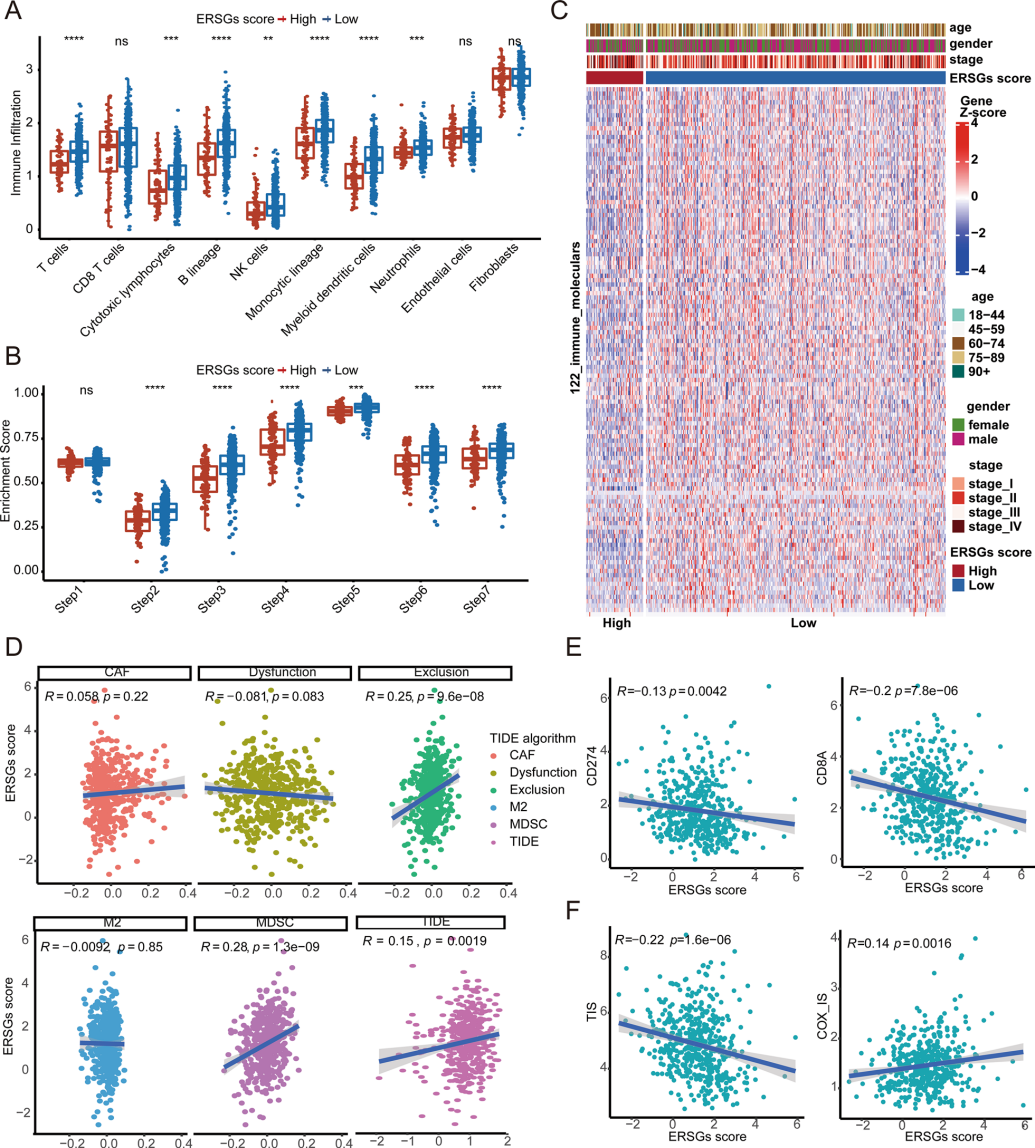
Relationships between ERSGs score and ICB response. **A** The distribution of immune cells infiltrating in the TME inferred by MCP-counter algorithm between the high and low ERSGs score groups in TCGA COAD cohort. **B** The boxplot shows the differences in enrichment scores of cancer immunity cycles calculated by ssGSEA between the high and low ERSGs score groups. **C** Heatmap shows the mRNA expressions of 122 immunomodulators between the high and low ERSGs score groups. **D** The Pearson correlation analysis between ERSGs scores and tumor-associated fibroblast (CAF), T cell dysfunction (Dysfunction), T cell exclusion (Exclusion), M2 macrophage (M2), myeloid-derived suppressor cell (MDSCs), and TIDE score. **E**, **F** The Pearson correlation analysis between ERSGs scores and ICB response predictors, including *CD8A*, *PD-L1* (*CD274*), TIS, and COS-IS.

To explore the relationships between ERSGs scoring system and tumor immunity, we designed the following analyses in the TCGA COAD cohort. First, MCP-counter and ssGSEA analyses showed that the abundance of most immune cells infiltrating in the TME were elevated in the low ERSGs score group, including CD8 T cells, activated CD4 and CD8 T cells, and natural killer cells (Fig. 6a, Additional file 8: Fig. S8B). Second, most steps of cancer immunity cycles (such as, cancer antigen presentation, priming and activation, trafficking of immune cells to tumors, and killing of cancer cells), as well as adaptive and innate immunity were more activated in the low ERSGs score groups than in the high ERSGs score group (Fig. 6b, Additional file 8: Fig. S8C). Third, patients with low ERSGs scores highly expressed most 122 immunomodulators, and inhibitory immune checkpoints, such as, *CD86*, *CD80*, and *TIGIT* (Fig. 5c, ICI). These findings revealed potential relationships between ERSGs scoring system and tumor immunity.

To analyze whether the ERSGs scoring system was associated with ICB therapy, we further explored the relationship between ERSGs score and several well-known ICB response predictors, such as tumor mutation burden (TMB) (45), MSI status (46), *PD-L1*, *CD8A*, TIDE score, TIS, and COX-IS. No significant difference was observed between the high and low ERSGs score groups in the TMB (Additional file 8: Fig. S8D). And the ERSGs scores between MSI-H and MSI-L/MSS patients were not significant different (Additional file 9: Fig. S9E). However, TIDE algorithm was used to predict ICB response based on transcriptome signatures, and showed that the ERSGs score was positively correlated with two immunosuppressive indices: T cell exclusion and MDSC (figure, T cell exclusion [R = 0.25, *P*-value = 9.6e−08], and MDSC [R =  − 0.28, *P*-value = 1.3e−09]). Of note, there was a strongly direct correlation between the ERSGs and TIDE scores (Fig. 6d, R = 0.15, *P*-value = 0.0019). Additionally, it is reported that patients with high expression of *PD-L1* and *CD8A* are more likely to benefit from ICB treatment. We observed that the ERSGs score was negatively correlated with the expression levels of *PD-L1* and *CD8A* (Fig. 6e, R =  − 0.13, *P*-value = 0.0042; R =  − 0.2, *P*-value = 7.8e−06). And the ERSGs scores were inversely and directly correlated with TIS (a positive ICB response predictor) and COX-IS (a negative ICB response predictor) (Fig. 6f).

Furthermore, the biomarker evaluation module on the TIDE website was used to assess the accuracy of the ERSGs scoring system as compared to other published biomarkers in multiple ICB treatment cohorts. The ERSGs scoring system displayed an AUC of more than 0.5 in 14 out of 16 ICB treatment cohorts when *CCL22*, *HOXB8*, *INHBB*, *KLK10*, *ZFP36*, and *ZNF703* serve as input genes (Additional file 9: Fig. S9), demonstrating its robustness as a predictive biomarker [65]. These findings indicated that the ERSGs score served as a potential predictor to reflect wore ICB therapy efficacy.

### ZNF703 severs as a promising target for tumor immunity

**Fig. 7:**
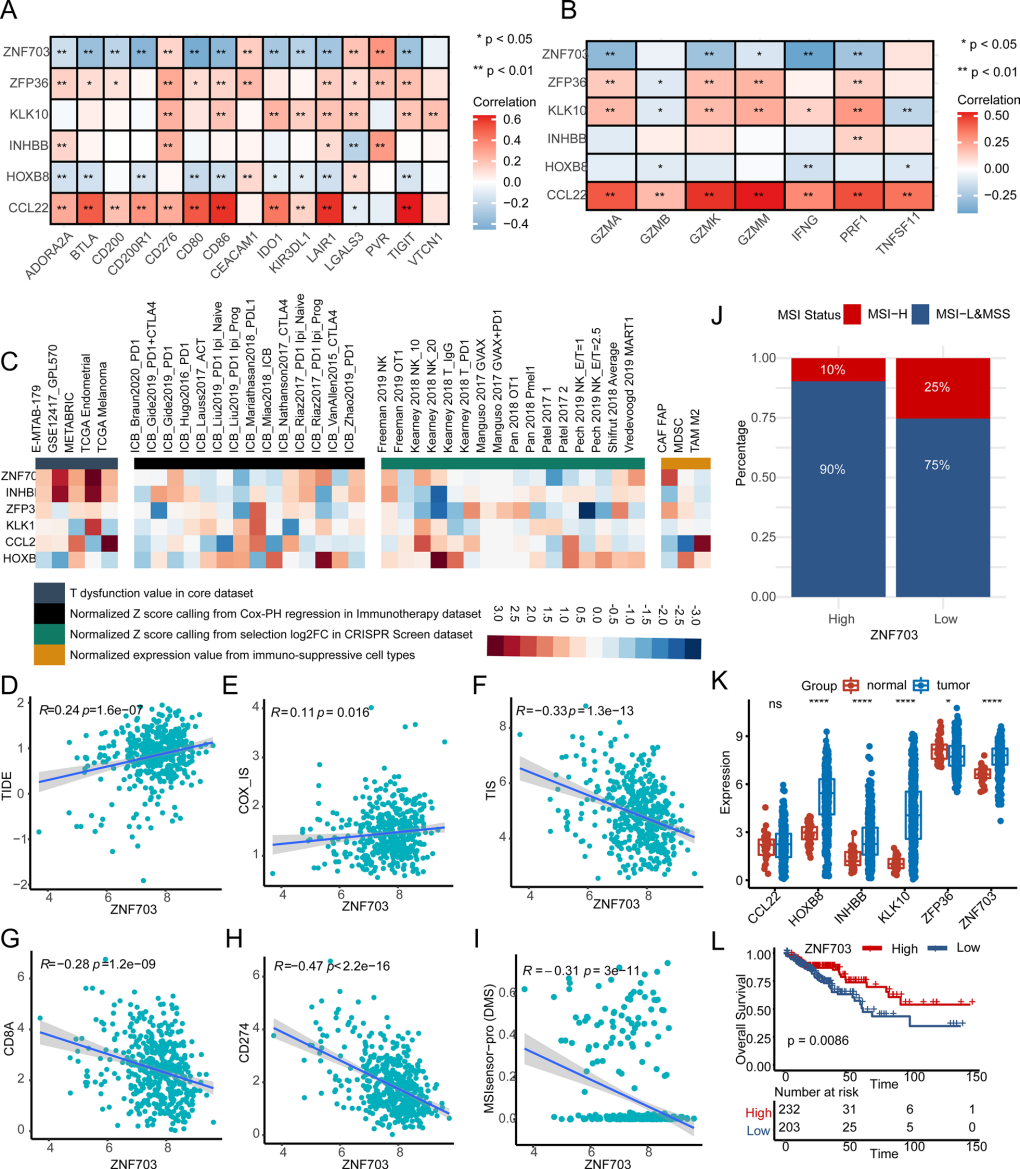
Exploring the biological functions of *ZNF703.* **A**, **B** The Pearson correlation between the six biomarker genes in ERSGs scoring system and immune checkpoint genes and cytotoxic genes (*GZMA*, *GZMB*, *GZMK*, *GZMM*, *IFNG*, *PRF1*, and *TNFSF11*) in TCGA COAD. Red and blue colors represent the positive and negative correlations, respectively. **C** The correlation between the six biomarker genes and four immunosuppressive indices (columns), including T cell dysfunction score (first column, T dysfunction value in core dataset), association with ICB survival outcome (second column, z-score in the Cox-PH regression in immunotherapy), log-fold change (logFC) in CRISPR screens (third column, helping identify regulators whose knockout can mediate the efficacy of lymphocyte-mediated tumor killing in cancer models), and T cell exclusion score (the fourth column, assessing the gene expression levels in immunosuppressive cell types that drive T cell exclusion). Genes (rows) are ranked by average value across four immunosuppressive indices analyzed using the TIDE website. **D**–**I** The Pearson correlation between *ZNF703* expression and TIDE score, COX-IS, TIS, *CD8A*, *PD-L1* (*CD274*), and MSI score in TCGA COAD cohort. **J** The stacked histogram shows the distribution of MSI-H and MSI-L/MSS patients in the high and low expression of *ZNF703* groups. **K** The mRNA expressions of the six biomarker genes between normal and cancer tissue in TCGA COAD cohort. **L** KM plot shows the OS analysis of the high and low expression of *ZNF703* groups in the TCGA COAD cohort.

**Fig. 8:**
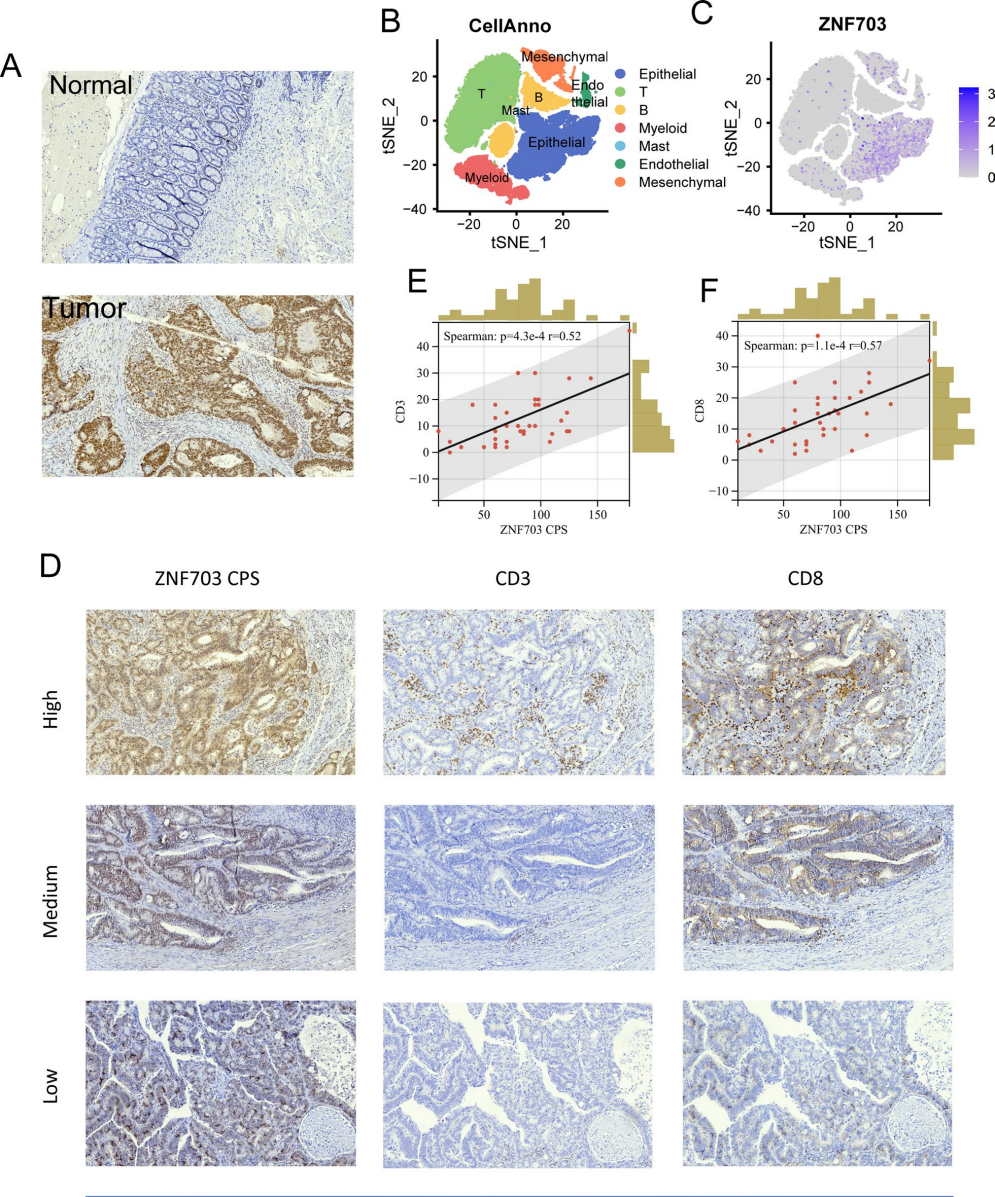
Exploring *ZNF703* in our IHC cohort. **A** Representative IHC images of *ZNF703* in CRC tissues (41 samples) and normal tissues (15 samples). **B**, **C** tSNE plots of 63,689 cells from 23 primary CRC samples and 10 matched normal mucosa samples, showing 7 clusters in each plot. Each cluster was shown in different color. Expression levels of ZNF703 illustrated in tSNE plot from both normal and tumor tissue in CRC patients. **D** Representative IHC images of ZNF703 CPS, CD3, and CD8 at different levels in the same view by consecutive sample sections. **E**, **F** The spearman correlation between ZNF703 CPS and the count of CD3 + and CD8 + T cells in proximity to ZNF703-staining cells

To further explore whether these biomarker genes (CCL22, HOXB8, INHBB, KLK10, ZFP36, and ZNF703) play a potential role in immune checkpoint therapy, we designed the following analyses. First, we analyzed the correlation between the biomarker genes and several immunological molecules, such as, immune checkpoint genes, and cytotoxic genes in TCGA COAD (Fig. 7a, b). Results demonstrated that most of the biomarker genes significantly correlated with immune checkpoint genes (BTLA, CD274, CTLA4, HAVCR2, LAG3, PDCD1, and TIGIT) and cytotoxic genes (GZMA, GZMB, GZMK, GZMM, IFNG, PRF1, and TNFSF11). Then, the regulator prioritization module on TIDE website that prioritizes genes with the best potential for developing combination immunotherapies showed that ZNF703 was the most potential target for mechanistic follow-up experiments, whose expression was positively correlated with T cell dysfunction phenotypes in all datasets enumerated (Fig. 7c, left panel). We also found that ZNF703 expression positively correlated with the biomarkers that predicted worse ICB response, including TIDE, and COX-IS scores (Fig. 7d, e); and negatively correlated with the biomarkers that predicted better ICB response, including TIS, CD8A, PD-L1, and MSI scores (Fig. 7f–i). We then established a HCT cell line with ZNF703 overexpression. We confirmed the successful transfection of ZNF703 through fluorescence, mRNA, and protein analyses (Additional file 10: Fig. S10A–C). Furthermore, we have also assessed the expression of PD-L1, and the results indicate that overexpression of ZNF703 in the HCT116 cell line did not lead to significant changes in PD-L1 protein expression (Additional file 10: Fig. S10C). Additionally, patients in MSI-H group expressed less ZNF703 than in MSI-L/MSS (Additional file 10: Fig. S10D), and the low ZNF703 expression group had a higher proportion of patients with MSI-H (Fig. 7j). The analysis results of CCL22, HOXB8, INHBB, KLK10, and ZFP36 were inferior to ZNF703 (Additional file 10: Fig. S10–Additional file 14: Fig. S14). Next, we analyzed the expression levels of the biomarker genes between normal and tumor tissues, and their prognostic roles. The expression values of *HOXB8*, *INHBB*, and *KLK10* were significantly upregulated in tumor tissues, while *ZFP36* were highly expressed in normal tissues, which were validated in paired tumor and normal tissues (Fig. 7k, S15A-D: TCGA COAD, GSE32323, GSE44076, GSE89076, and GSE113513). KM analysis showed that patients with high expression of *CCL22* exhibited better OS, and patients with high expression of *INHBB* exhibited worse OS (Additional file 15: Fig. S15E–I). Of note, *ZNF703* expression was significantly upregulated in tumor tissues as compared to normal tissues in TCGA COAD cohort (Fig. 6k), and verified in GSE32323, GSE44076, GSE89076, and GSE113513 (Additional file 15: Fig. S15A–D). And KM analysis showed that patients with high expression of *ZNF703* exhibited better OS (Fig. 7l). Based on mRNA analysis, patients with low ZNF703 expression exhibited poorer survival; however, this subgroup of patients might potentially display heightened sensitivity to ICB therapy. Additionally, we performed IHC analysis on tissue sections from 56 CRC patients to delve into the relationship between ZNF703 and tumor immunity at the protein level. Remarkably, our results revealed a significant upregulation of ZNF703 in CRC tissues as compared to normal tissues (Fig. 8a). Additionally, ZNF703 was predominantly expressed in epithelial cells, consistent with our analysis of single-cell and spatial transcriptomic data (Fig. 8b, c). Furthermore, IHC analysis of consecutive sections of CRC tissues demonstrated a significant correlation between the number of ZNF703-positive cells and the infiltration of CD3 + T cells and CD8 + T cells (Fig. 8d–f, Additional file 30: Table S15). Collectively, these compelling results underscore the substantial impact of tumor cell-expressed ZNF703 on tumor immunity.

## Discussion

Although ERS plays an important role in various biological processes of tumor [66], such as autophagy [67], angiogenesis [68], metastasis [69, 70], the role of ERS in CRC and tumor immunity has not been fully elucidated.

In this study, we identified two ERS clusters based on our collected ERS genes in CRC (defined as ERS clusters 1 and 2). Key genes between the ERS clusters, identified by WGCNA analysis, identify another two clusters again (defined as ERSGs clusters 1 and 2). Significant differences were found in gene expression patterns, OS, and immune status in ERS clusters and ERSGs clusters. Compared with ERS cluster 1 and ERSGs cluster 1, patients in ERS cluster 2 and ERSGs cluster 2 exhibited worse survival, and active immune status. Additionally, Angio markers highly expressed in ERS Cluster 2, which may be one of the mechanisms responsible for the poor prognosis and indicated that patients in ERS cluster 2 may be more likely to benefit from anti-angiogenic drugs.

Our study demonstrated that the ERSGs scoring system is a robust prognostic model. First, KM analysis in multiple CRC cohorts demonstrated that patients with high ERSGs scores exhibited worse OS, RFS, DFS and DSS than patients with low ERSGs scores. Second, univariate and multivariate Cox analyses demonstrated that scoring system was an independent risk factor associated with OS after adjusting for age, gender, and stage. Third, subgroup analysis also demonstrated the prognostic value of the scoring system in young, old, male, female, and stage III&IV in the combined GEO, GSE39582, and TCGA COAD cohorts. The prognostic value of the scoring system was not significant in the stage I&II in TCGA COAD cohort, which may suggest that the scoring system has greater prognostic power in advanced (stage III&IV) CRC patients. Finally, according to the ROC analyses from the combined GEO, GSE39582, and TCGA COAD datasets, our model demonstrated promising capabilities in prognosticating short-term patient survival, notably 1-year survival.

Additionally, our study showed that patients with low ERSGs score may be more likely to benefit from ICI therapy. According to previous studies [71, 72], we defined patients with low ERSGs scores as “hot” tumors characterized by an increased infiltration of CD4 and CD8 T cells to the TME, and patients with high ERSGs scores as “cold” tumors characterized by the absence or low presence of lymphocytes in the TME. Studies have shown that “hot” tumors are more sensitive to ICB therapy than “cold” tumors [71, 72]. Consistently, the ERSGs scores are negatively correlated with multiple positive ICB response predictors (*CD8A*, *PD-L1*, and TIS) [53, 73], and positively correlated with multiple negative ICB response predictors (TIDE, T cell Exclusion, COX-IS) [52, 54, 65]. Therefore, we speculate that the prognosis of patients with low ERSGs score may improve under ICB therapy.

Of note, we identified *ZNF703* as a promising target for immunotherapies. At the mRNA level, patients with low *ZNF703* expression exhibited poorer survival; however, this subgroup potentially displayed heightened sensitivity to ICB therapy. Zinc finger protein 703 (*ZNF703*), a member of the zinc finger transcription factor Net/NLZ family, abnormally highly expressed in liver cancer [74], ovarian cancer [75], breast cancer [76], and head and neck squamous cell carcinoma [77]. The abnormal expression of *ZNF703* affects the biological behavior of tumor cells and the prognosis of patients. And there is one study reported that mRNA and protein levels of *ZNF703* were upregulated in CRC tissues than normal mucosal tissues, and patients with high protein levels of *ZNF703* exhibited poor cancer-specific survival [78]. In our investigation, *ZNF703* exhibited elevated expression levels within tumor tissues, concurrently demonstrating a favorable prognostic association at the mRNA level. Additionally, *ZNF703* CPS showed a positive correlation with CD8 + T cell abundance in our IHC cohort, contrasting with an inverse correlation at the mRNA level in TCGA COAD cohort. The inconsistent effects of *ZNF703* at mRNA and protein levels potentially arise from mRNA post-transcriptional modification, ribosomal translation efficiency and post-translational modifications of proteins. In the future, elucidating this phenomenon will require RNA-seq, ribosome profiling, proteomics analysis, and larger sample sizes to provide comprehensive insights.

There are still some inherent limitations in our study. Owing to the absence of transcriptome data related to ICB therapy in CRC, the discriminative efficacy of the ERSGs score and *ZNF703* in CRC patients could not be substantiated. Nevertheless, we intend to further corroborate their validity once public ICB transcriptome data for CRC becomes accessible. And we plan to collect CRC tumor tissues and transcriptomic data from patients undergoing immunotherapy in a CRC retrospective ICB cohort or prospectively in a trial. Through rigorous analysis of treatment responses and survival outcomes, we aim to comprehensively assess the potential clinical applicability of the scoring system and *ZNF703*. And functional experiments for validation of the ZNF703 as a tumor immunity target are very important. One experiment that can be carried out is to knock out/over express ZNF703 in a CRC cell line and look at the effect on the viability of these modified cells vs control cells when co-cultured with T-cells.

## Conclusions

In conclusion, our study demonstrated that different ERS statuses (ERS clusters, and ERSGs clusters) are closely related to the prognoses and immune statuses of CRC patients. And ERSGs scoring system can serve as an effective model to discriminate the outcomes of CRC patients. Patients with low *ZNF703* mRNA expression potentially benefit from ICB therapy. Moreover, we identified *ZNF703* as a promising target for tumor immunity.

### Supplementary Information

**Additional file 1. Fig. S1**: Processes of constructing ERS clusters. **A**–**C** Consensus matrixes of the combined GEO cohort for each k (k = 2–4), displaying the clustering stability using 1000 iterations of hierarchical clustering. **D** Empirical cumulative distribution function plot displays consensus distributions for each k. When k = 2, the distribution reaches an approximate maximum, indicating maximum stability. **E** The distribution of immune cells infiltrating in the TME inferred by MCP-counter algorithm between ERS clusters. **F** Enrichment scores for adaptive and innate immunity between ERS clusters, as deduced through ssGSEA analysis. **G**, **H** The proportion of different CMS patients in ERS clusters. The statistical differences between the two groups will be assessed using a chi-square test.

**Additional file 2. Fig. S2**: Validating ERS Classification Robustness in TCGA COAD cohort. **A** The PCA diagram shows the different gene expression patterns between ERS clusters. **B** KM plot shows the OS analysis of ERS clusters. **C** The heatmap reveals the relationships between ERS clusters and 11 critical biological pathways. **D** The difference in mRNA expression of 20 inhibitory immune checkpoints between the ERS clusters. **E** The boxplot shows the differences in enrichment scores of cancer immunity cycles calculated by ssGSEA between ERS clusters. **F** The distribution of 28 types of immune cells infiltration between ERS clusters inferred by ssGSEA analysis.

**Additional file 3. Fig. S3**: Details of the WGCNA analysis. **A**, **B** Analysis of the scale-free fit index and the mean connectivity for various soft-thresholding power values. **C** Hierarchical clustering dendrograms of co-expressed genes in modules. **D**, **E** The correlation between modules. **F**–**H** The correlation between module eigengenes and ERS cluster 1 in blue, brown, and green modules.

**Additional file 4. Fig. S4**: GO and KEGG analyses of key WGCGA modules. **A**, **B** The top 15 GO and KEGG enrichment terms in blue, yellow, and green modules, respectively. An adjusted *P*-value of less than 0.05 was regarded as statistically significant.

**Additional file 5. Fig. S5**: Details of constructing ERSGs clusters. **A**–**D** Consensus matrixes in the combined GEO cohort for each k (k = 2–5), displaying the clustering stability using 1000 iterations of hierarchical clustering. **E** Empirical cumulative distribution function plot displays consensus distributions for each k. When k = 2, the distribution reaches an approximate maximum, indicating maximum stability. the distribution reaches an approximate maximum, indicating maximum stability.

**Additional file 6. Fig. S6**: Exploring the biological functions between ERSGs clusters. **A** The heatmap reveals the relationships between ERSGs clusters and 11 critical biological pathways. Rows of the heat map represent gene expression grouped by pathway. Red and blue colors represent high and low expression, respectively. **B** Heatmap shows the mRNA expressions of 122 immunomodulators between the ERSGs clusters. **C** The distribution of 28 types of immune cells infiltration between ERSGs clusters inferred by ssGSEA analysis.

**Additional file 7. Fig. S7**: Exploring the clinical role of ERSGs scoring system. **A**, **B** Details of the Lasso regression in the combined GEO cohort. **C** The survival analysis of ERSGs scores in multiple CRC cohorts. RFS represents recurrence-free survival, DFS represents disease-free survival, and DSS represents disease-specific survival. **D** Multivariable Cox regression analysis of OS in GSE39582 cohort. **E** Subgroup survival analysis of ERSGs scoring system in different age, gender, and TNM stages in GSE39582 cohort.

**Additional file 8. Fig. S8**: Exploring the role of ERSGs scoring system in TCGA COAD. **A** The distribution of ERSGs scores in different CMS groups. **B** The distribution of 28 types of immune cells infiltration between the high and low ERSGs score groups inferred by ssGSEA analysis. **C** The differences of enrichment scores of adaptive and innate immunity between the high and low ERSGs score groups inferred by ssGSEA analysis. **D** The levels of TMB between the high and low ERSGs score groups. **E** The distribution of ERSGs scores between MSI-H and MSI-L/MSS patients. The statistic differences between two groups are assessed by the Wilcox test.

**Additional file 9. Fig. S9**: Comparison of the power predicting ICB response in ERSGs scoring system and other biomarkers. AUC is used to evaluate the predictive performance of the ERSGs scoring system (Custom) and other biomarkers on ICB response in 16 ICB treatment cohorts on the TIDE website.

**Additional file 10. Fig. S10**: Exploring the biological functions of CCL22. **A** Representative fluorescence images after plasmid transfection for 24–48 h. **B** Quantitative real-time PCR analysis was conducted to assess the overexpression of ZNF703 at 24–48 h post-plasmid transfection. OE: overexpression, NC: normal control, OE-ZNF703-4: cells transfected with 4 μL Lipo8000, and OE-ZNF703-5: cells transfected with 5 μL Lipo8000. Subsequent experiments involved cell transfection using 5 μL Lipo8000. **C** Western blotting was employed to evaluate the efficiency of ZNF703 overexpression and the expression levels of PDL1. Untreated: untreated HCT116 cells. **D** The expression of ZNF703 between MSI-H and MSI-L/MSS patients. **E**–**J** The Pearson correlation between CCL22 expression and TIDE score, COX-IS, TIS, CD8A, PD-L1 (CD274), and MSI score in TCGA COAD cohort. **K** The expression of CCL22 between MSI-H and MSI-L/MSS patients. **L** The stacked histogram shows the distribution of MSI-H and MSI-L/MSS patients in the high and low expression of CCL22 groups.

**Additional file 11. Fig. S11**: Exploring the biological functions of HOXB8 in ERSGs scoring system. **A**–**F** The Pearson correlation between HOXB8 expression and TIDE score, COX-IS, TIS, CD8A, PD-L1 (CD274), and MSI score in TCGA COAD cohort. **G** The expression of HOXB8 between MSI-H and MSI-L/MSS patients. **H** The stacked histogram shows the distribution of MSI-H and MSI-L/MSS patients in the high and low expression of HOXB8 groups.

**Additional file 12. Fig. S12**: Exploring the biological functions of INHBB in ERSGs scoring system. **A**–**F** The Pearson correlation between INHBB expression and TIDE score, COX-IS, TIS, CD8A, PD-L1 (CD274), and MSI score in TCGA COAD cohort. **G** The expression of INHBB between MSI-H and MSI-L/MSS patients. **H** The stacked histogram shows the distribution of MSI-H and MSI-L/MSS patients in the high and low expression of INHBB groups.

**Additional file 13. Fig. S13**: Exploring the biological functions of KLK10 in ERSGs scoring system. **A**–**F** The Pearson correlation between KLK10 expression and TIDE score, COX-IS, TIS, CD8A, PD-L1 (CD274), and MSI score in TCGA COAD cohort. **G** The expression of KLK10 between MSI-H and MSI-L/MSS patients. **H** The stacked histogram shows the distribution of MSI-H and MSI-L/MSS patients in the high and low expression of KLK10 groups.

**Additional file 14. Fig. S14**: Exploring the biological functions of ZFP36 in ERSGs scoring system. **A**–**F** The Pearson correlation between ZFP36 expression and TIDE score, COX-IS, TIS, CD8A, PD-L1 (CD274), and MSI score in TCGA COAD cohort. **G** The expression of ZFP36 between MSI-H and MSI-L/MSS patients. **H** The stacked histogram shows the distribution of MSI-H and MSI-L/MSS patients in the high and low expression of ZFP36 groups.

**Additional file 15. Fig. S15**: Expression levels and survival analysis of biomarker genes in ERSGs scoring system. **A**–**D** The mRNA expressions of the six biomarker genes in ERSGs scoring system between normal and cancer tissue in GSE32323, GSE44076, GSE89076, and GSE113513 cohorts. **E**–**I** KM plots show the OS analysis of the high and low expression of CCL22, HOXB8, INHBB, KLK10, and ZFP36 groups in the TCGA COAD cohort.

**Additional file 16. Table S1**: Detail information of public CRC cohorts used in this study.

**Additional file 17. Table S2**: ERS related genes identified in this study.

**Additional file 18. Table S3**: 122 immune molecules collected from previous studies.

**Additional file 19. Table S4**: 20 inhibitory immune checkpoints.

**Additional file 20. Table S5**: Survival data used for survival analysis.

**Additional file 21. Table S6**: TIS genes.

**Additional file 22. Table S7**: COX-IS genes.

**Additional file 23. Table S8**: MSI status of patients in TCGA COAD inferred by MSIsensor pro.

**Additional file 24. Table S9**: Multivariate cox regression of ERS clusters.

**Additional file 25. Table S10**: WGCNA module genes.

**Additional file 26. Table S11**: GO and KEGG results of different WGCNA modules.

**Additional file 27. Table S12**: Genes for constructing ERSGs scoring system.

**Additional file 28. Table S13**: Coefficients of genes in ERSGs.

**Additional file 29. Table S14**: CMS groups inferred by CMScaller.

**Additional file 30. Table S15**: IHC scores of CRC samples.

## Data Availability

The original data presented in the study are included in GEO and TCGA websites (Additional file 16: Table S1).

## Abbreviations

APM: Antigen processing machinery
CD8 + Teff: CD8 T-effector signature
CMS: Consensus Molecular Subtypes
CRC: Colorectal cancer
DDR: DNA damage repair
DFS: Disease-free survival
DSS: Disease-specific survival
EMT: Epithelial-mesenchymal transition
ERS: Endoplasmic reticulum stress
ERSGs: Endoplasmic reticulum stress-related genes
F-TBRS: Pan-tissue fibroblast TGF-β response
GO: Gene Ontology
ICB: Immune checkpoint blockade
ICI: Immune checkpoint signature
KEGG: Kyoto Encyclopedia of Genes and Genomes
KM: Kaplan–Meier
MCP-counter: Microenvironment Cell Populations-counter
MHC: Major histocompatibility complex
MSigDB: Molecular Signatures Database
OS: Overall survival
RFS: Recurrence-free survival
ssGSEA: Single-sample gene set enrichment analysis
TME: Tumor microenvironment
TIDE: Tumor Immune Dysfunction and Exclusion
TIP: Tracking tumor immune phenotype
UPR: Unfolded protein response
WGCNA: Weighted Gene Co-expression Network Analysis
