# Supplementary figures and images for: Characterization of endoplasmic reticulum stress unveils ZNF703 as a promising target for colorectal cancer immunotherapy
Source: J Transl Med. 2023 Oct 11;21:713. doi: 10.1186/s12967-023-04547-z (PMC10566095; doi:10.1186/s12967-023-04547-z)

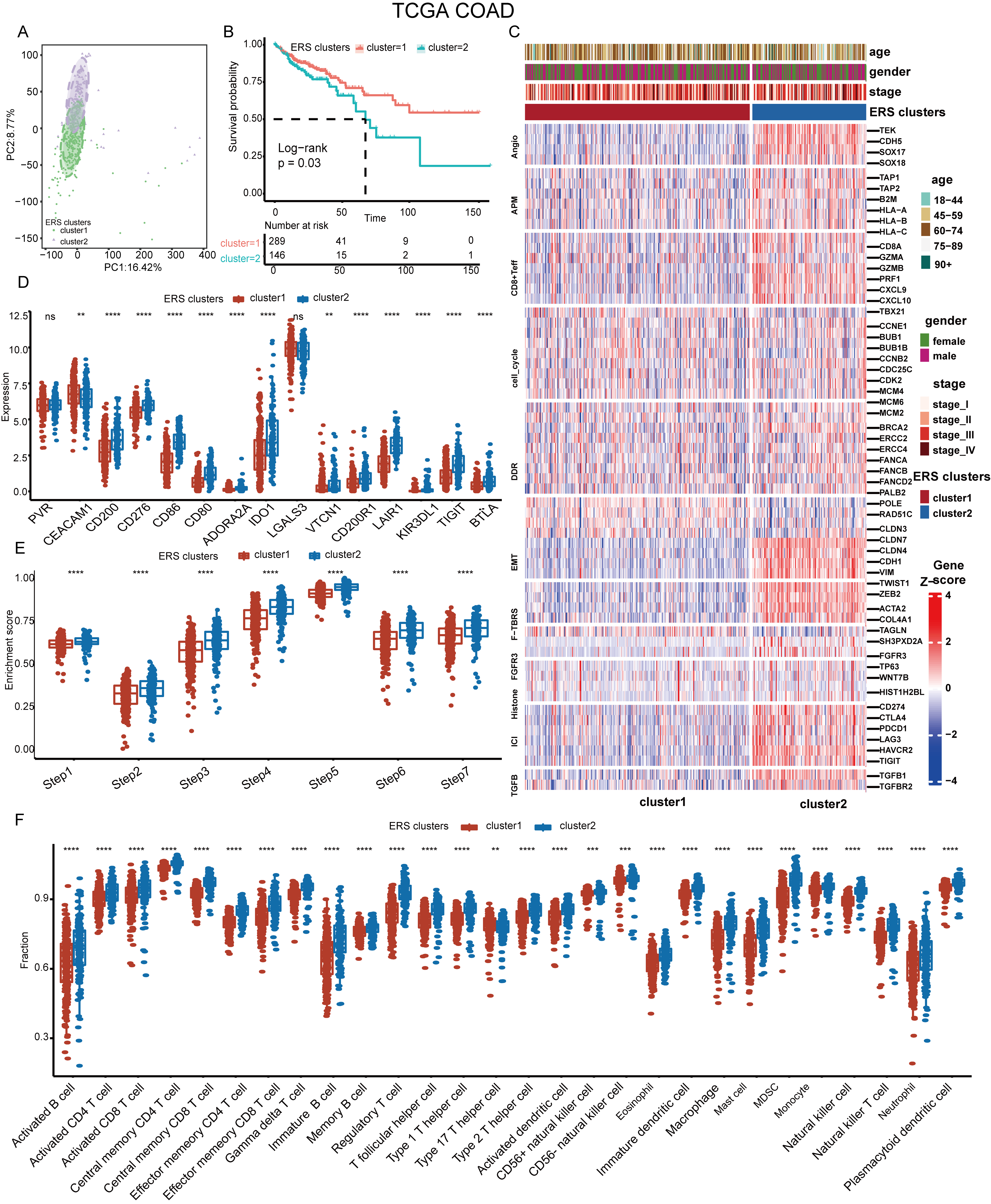

Supplement: Supplementary file 2 — Additional file 2. Fig. S2: Validating ERS Classification Robustness in TCGA COAD cohort. A The PCA diagram shows the different gene expression patterns between ERS clusters. B KM plot shows the OS analysis of ERS clusters. C The heatmap reveals the relationships between ERS clusters and 11 critical biological pathways. D The difference in mRNA expression of 20 inhibitory immune checkpoints between the ERS clusters. E The boxplot shows the differences in enrichment scores of cancer immunity cycles calculated by ssGSEA between ERS clusters. F The distribution of 28 types of immune cells infiltration between ERS clusters inferred by ssGSEA analysis. [file 12967_2023_4547_MOESM2_ESM.tif]

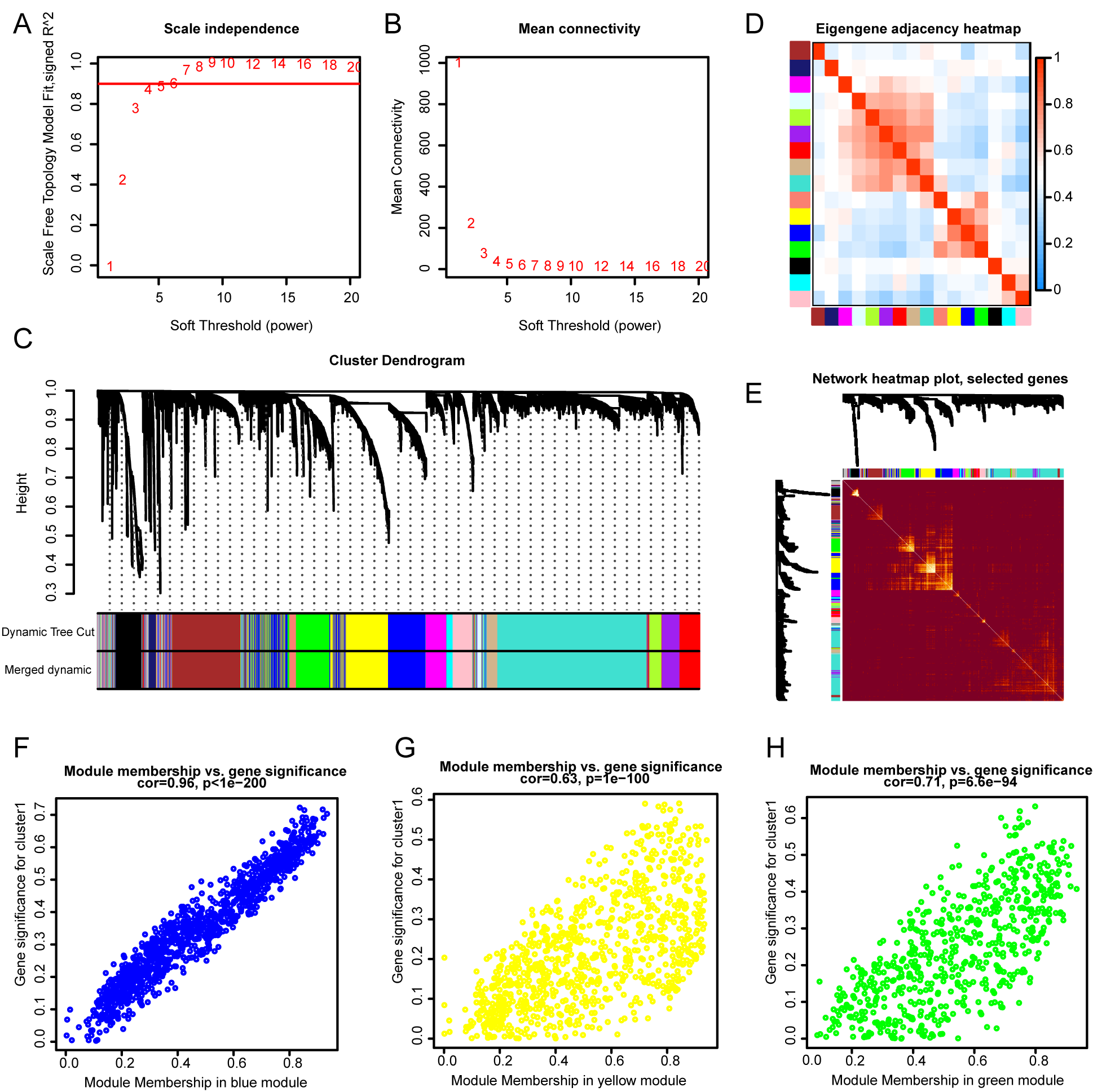

Supplement: Supplementary file 3 — Additional file 3. Fig. S3: Details of the WGCNA analysis. A, B Analysis of the scale-free fit index and the mean connectivity for various soft-thresholding power values. C Hierarchical clustering dendrograms of co-expressed genes in modules. D, E The correlation between modules. F–H The correlation between module eigengenes and ERS cluster 1 in blue, brown, and green modules. [file 12967_2023_4547_MOESM3_ESM.tif]

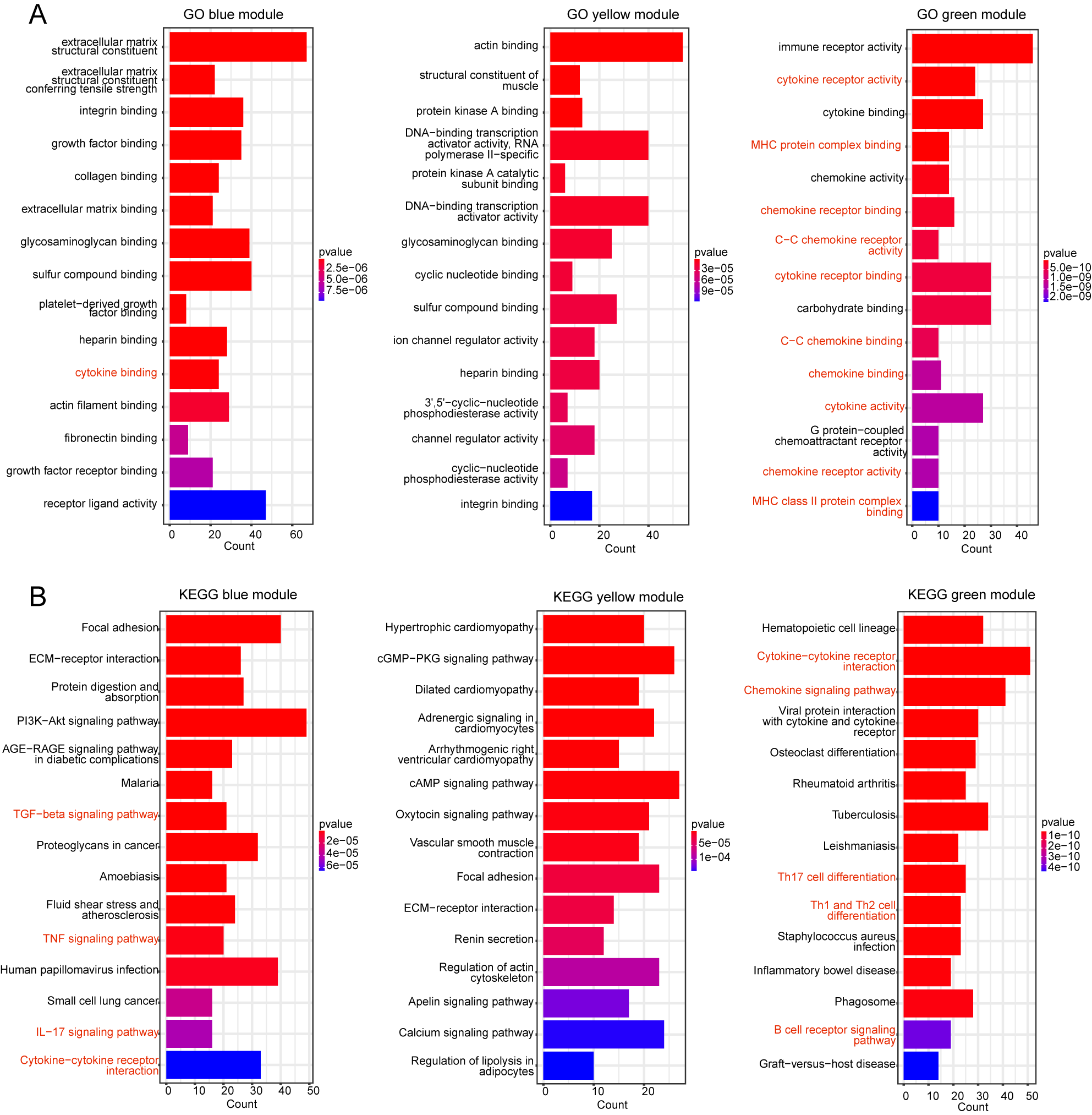

Supplement: Supplementary file 4 — Additional file 4. Fig. S4: GO and KEGG analyses of key WGCGA modules. A, B The top 15 GO and KEGG enrichment terms in blue, yellow, and green modules, respectively. An adjusted P-value of less than 0.05 was regarded as statistically significant. [file 12967_2023_4547_MOESM4_ESM.tif]

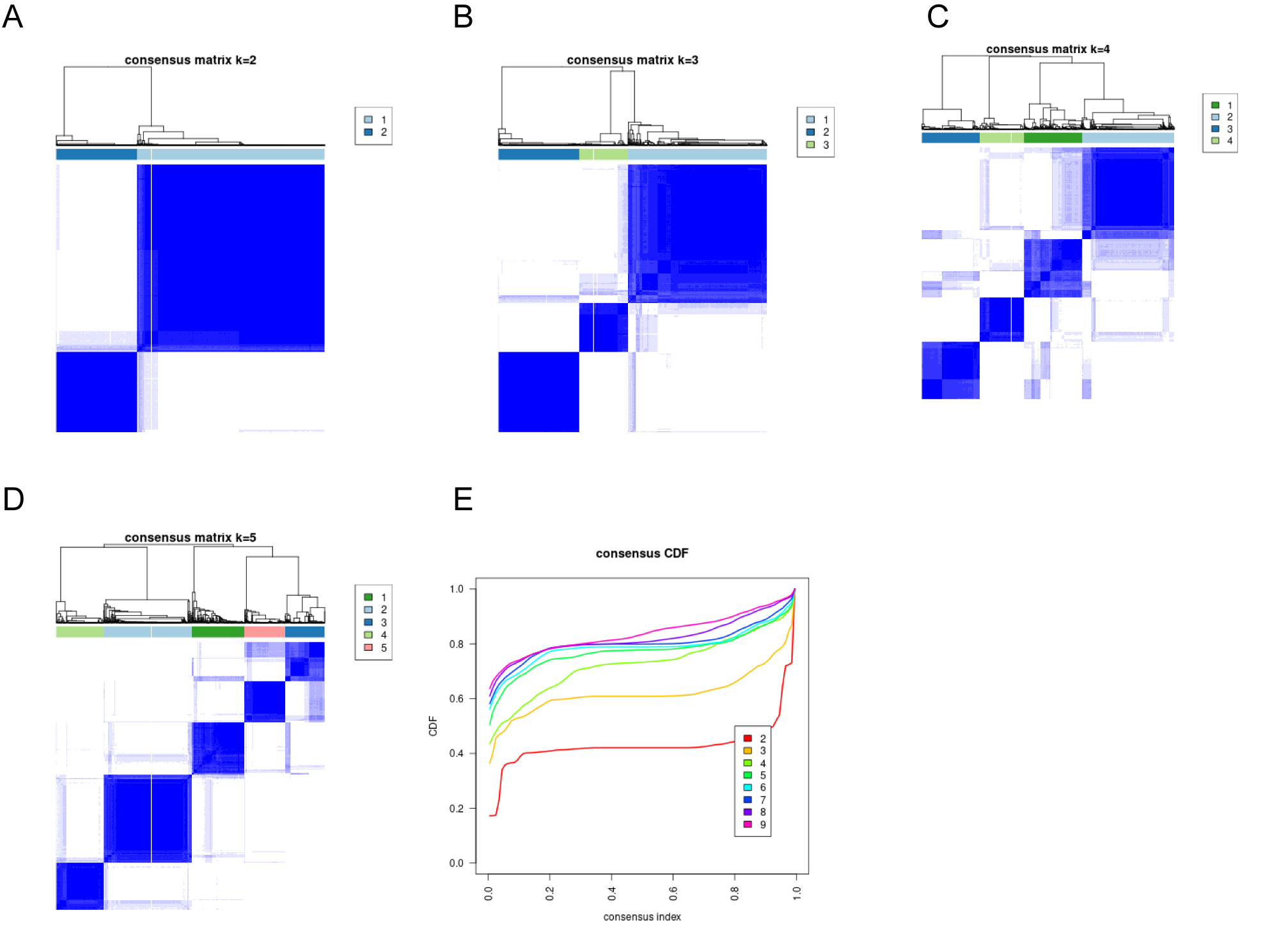

Supplement: Supplementary file 5 — Additional file 5. Fig. S5: Details of constructing ERSGs clusters. A–D Consensus matrixes in the combined GEO cohort for each k (k = 2–5), displaying the clustering stability using 1000 iterations of hierarchical clustering. E Empirical cumulative distribution function plot displays consensus distributions for each k. When k = 2, the distribution reaches an approximate maximum, indicating maximum stability. the distribution reaches an approximate maximum, indicating maximum stability. [file 12967_2023_4547_MOESM5_ESM.tif]

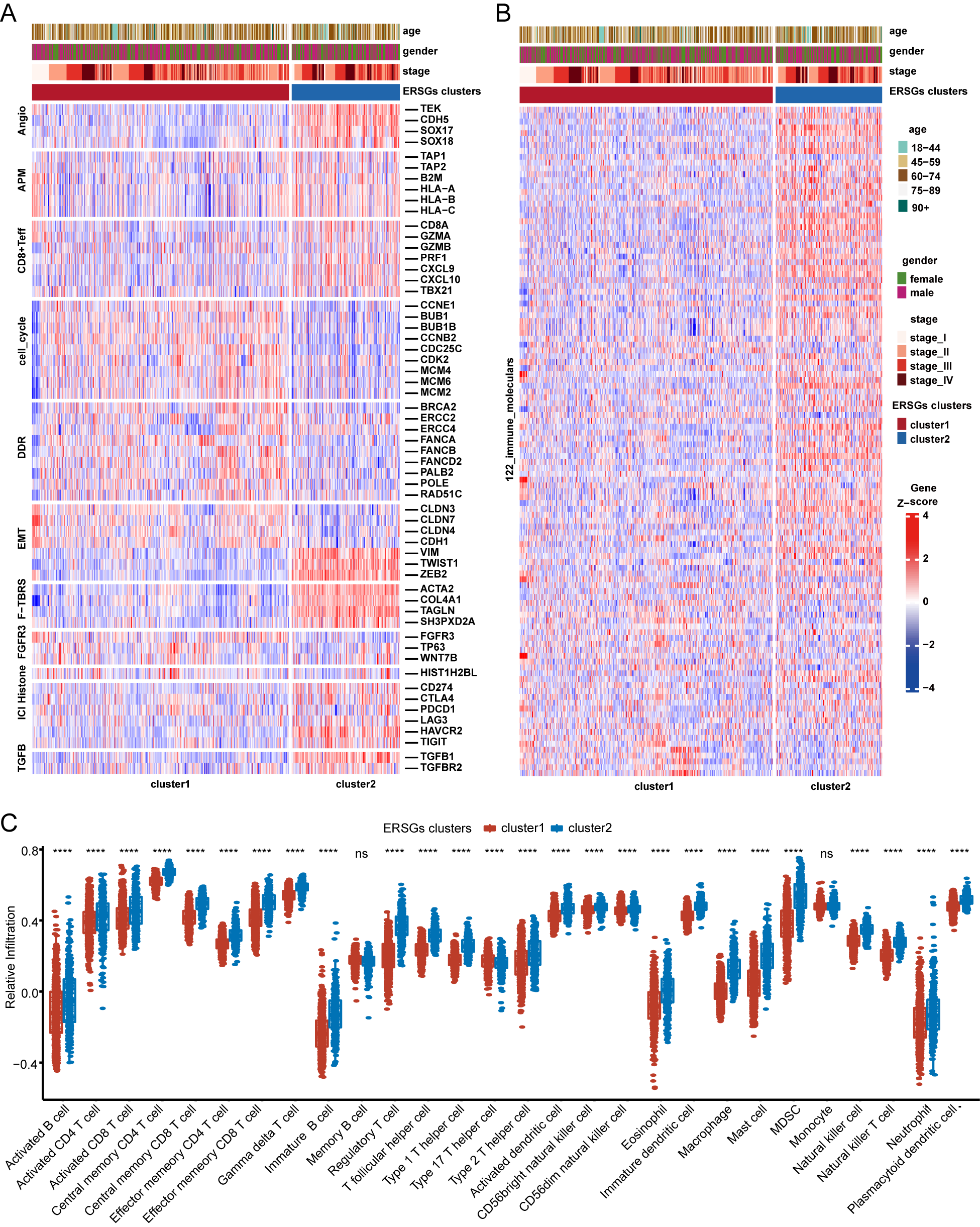

Supplement: Supplementary file 6 — Additional file 6. Fig. S6: Exploring the biological functions between ERSGs clusters. A The heatmap reveals the relationships between ERSGs clusters and 11 critical biological pathways. Rows of the heat map represent gene expression grouped by pathway. Red and blue colors represent high and low expression, respectively. B Heatmap shows the mRNA expressions of 122 immunomodulators between the ERSGs clusters. C The distribution of 28 types of immune cells infiltration between ERSGs clusters inferred by ssGSEA analysis. [file 12967_2023_4547_MOESM6_ESM.tif]

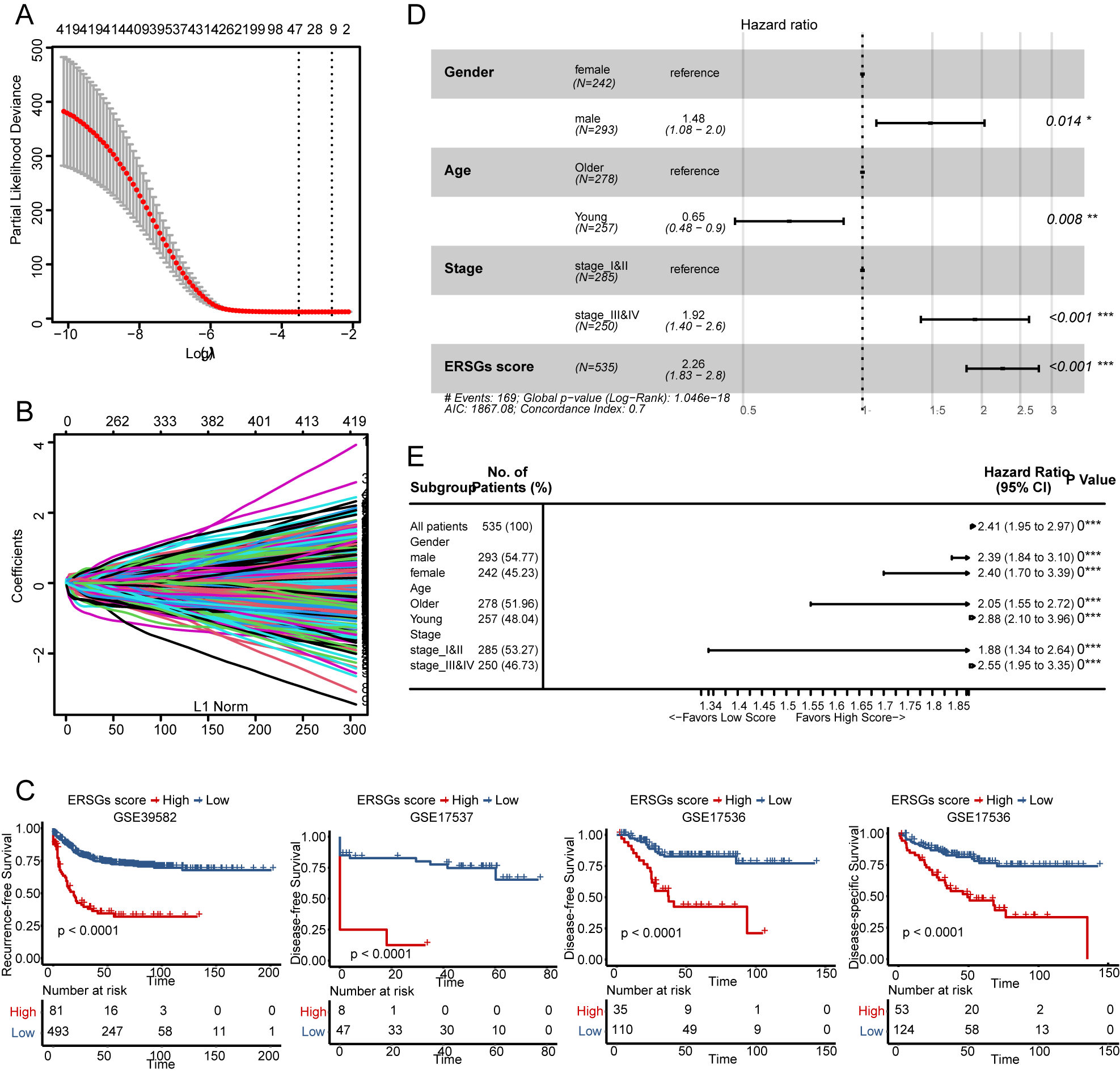

Supplement: Supplementary file 7 — Additional file 7. Fig. S7: Exploring the clinical role of ERSGs scoring system. A, B Details of the Lasso regression in the combined GEO cohort. C The survival analysis of ERSGs scores in multiple CRC cohorts. RFS represents recurrence-free survival, DFS represents disease-free survival, and DSS represents disease-specific survival. D Multivariable Cox regression analysis of OS in GSE39582 cohort. E Subgroup survival analysis of ERSGs scoring system in different age, gender, and TNM stages in GSE39582 cohort. [file 12967_2023_4547_MOESM7_ESM.tif]

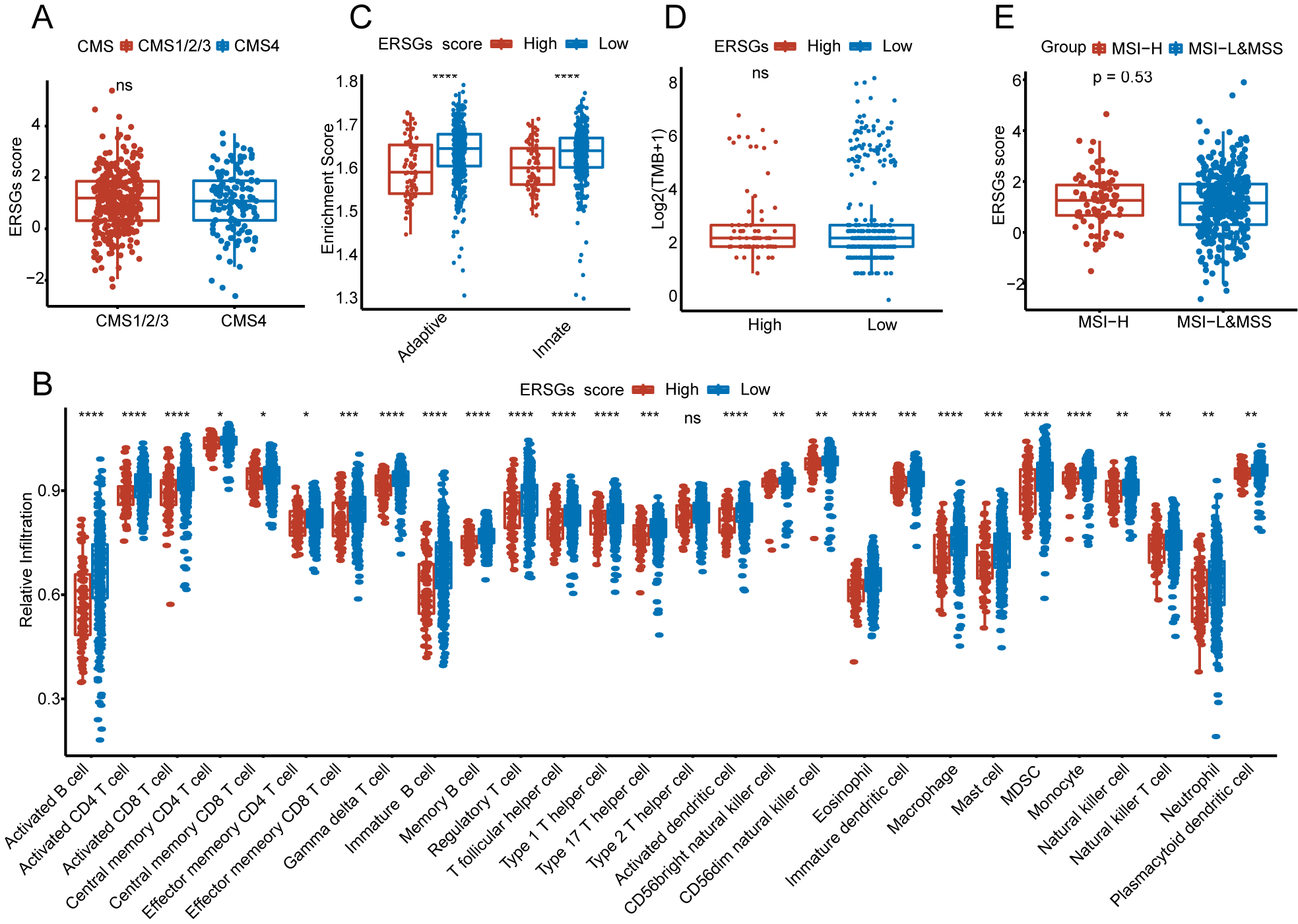

Supplement: Supplementary file 8 — Additional file 8. Fig. S8: Exploring the role of ERSGs scoring system in TCGA COAD. A The distribution of ERSGs scores in different CMS groups. B The distribution of 28 types of immune cells infiltration between the high and low ERSGs score groups inferred by ssGSEA analysis. C The differences of enrichment scores of adaptive and innate immunity between the high and low ERSGs score groups inferred by ssGSEA analysis. D The levels of TMB between the high and low ERSGs score groups. E The distribution of ERSGs scores between MSI-H and MSI-L/MSS patients. The statistic differences between two groups are assessed by the Wilcox test. [file 12967_2023_4547_MOESM8_ESM.tif]

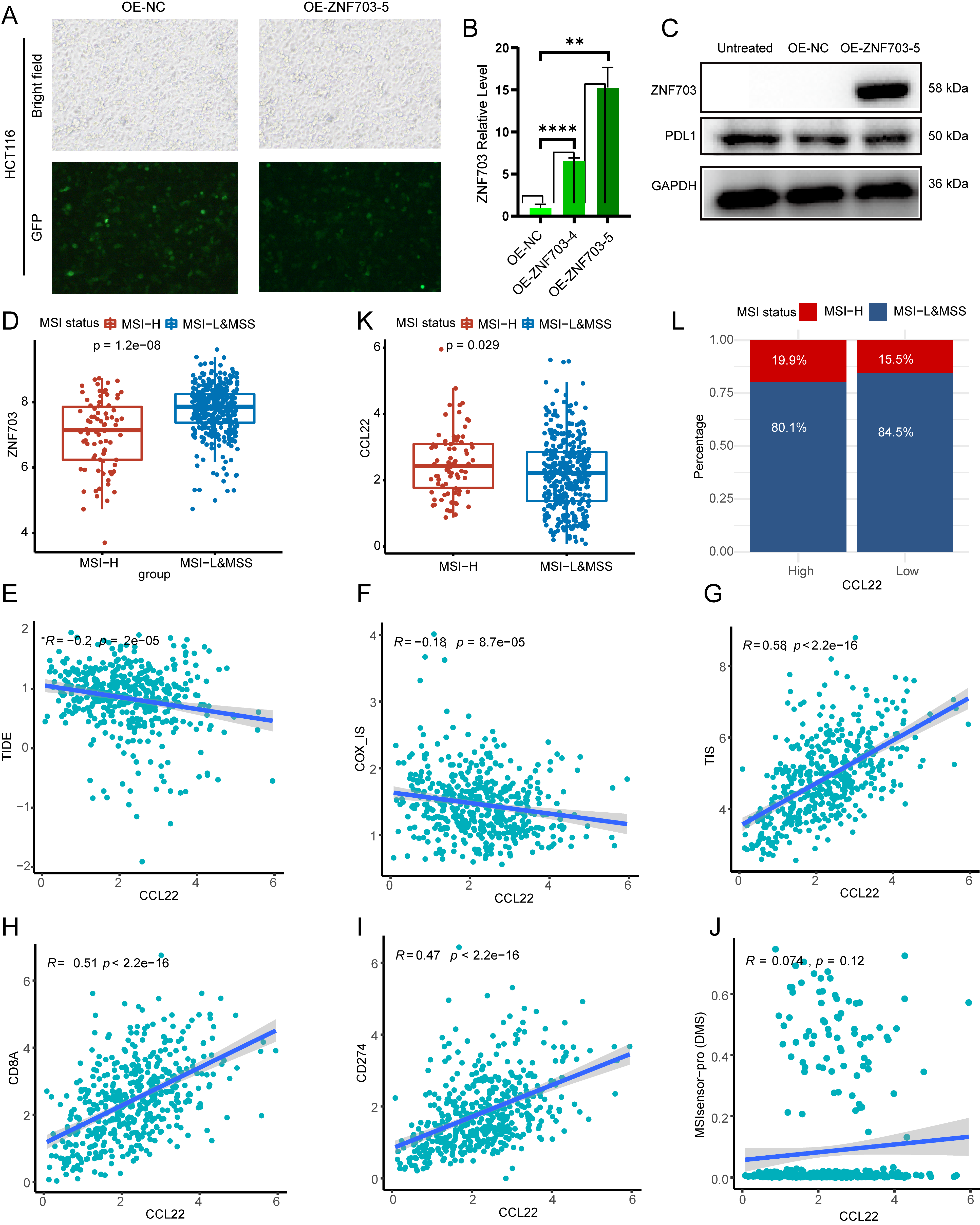

Supplement: Supplementary file 10 — Additional file 10. Fig. S10: Exploring the biological functions of CCL22. A Representative fluorescence images after plasmid transfection for 24–48 h. B Quantitative real-time PCR analysis was conducted to assess the overexpression of ZNF703 at 24–48 h post-plasmid transfection. OE: overexpression, NC: normal control, OE-ZNF703-4: cells transfected with 4 μL Lipo8000, and OE-ZNF703-5: cells transfected with 5 μL Lipo8000. Subsequent experiments involved cell transfection using 5 μL Lipo8000. C Western blotting was employed to evaluate the efficiency of ZNF703 overexpression and the expression levels of PDL1. Untreated: untreated HCT116 cells. D The expression of ZNF703 between MSI-H and MSI-L/MSS patients. E–J The Pearson correlation between CCL22 expression and TIDE score, COX-IS, TIS, CD8A, PD-L1 (CD274), and MSI score in TCGA COAD cohort. K The expression of CCL22 between MSI-H and MSI-L/MSS patients. L The stacked histogram shows the distribution of MSI-H and MSI-L/MSS patients in the high and low expression of CCL22 groups. [file 12967_2023_4547_MOESM10_ESM.tif]

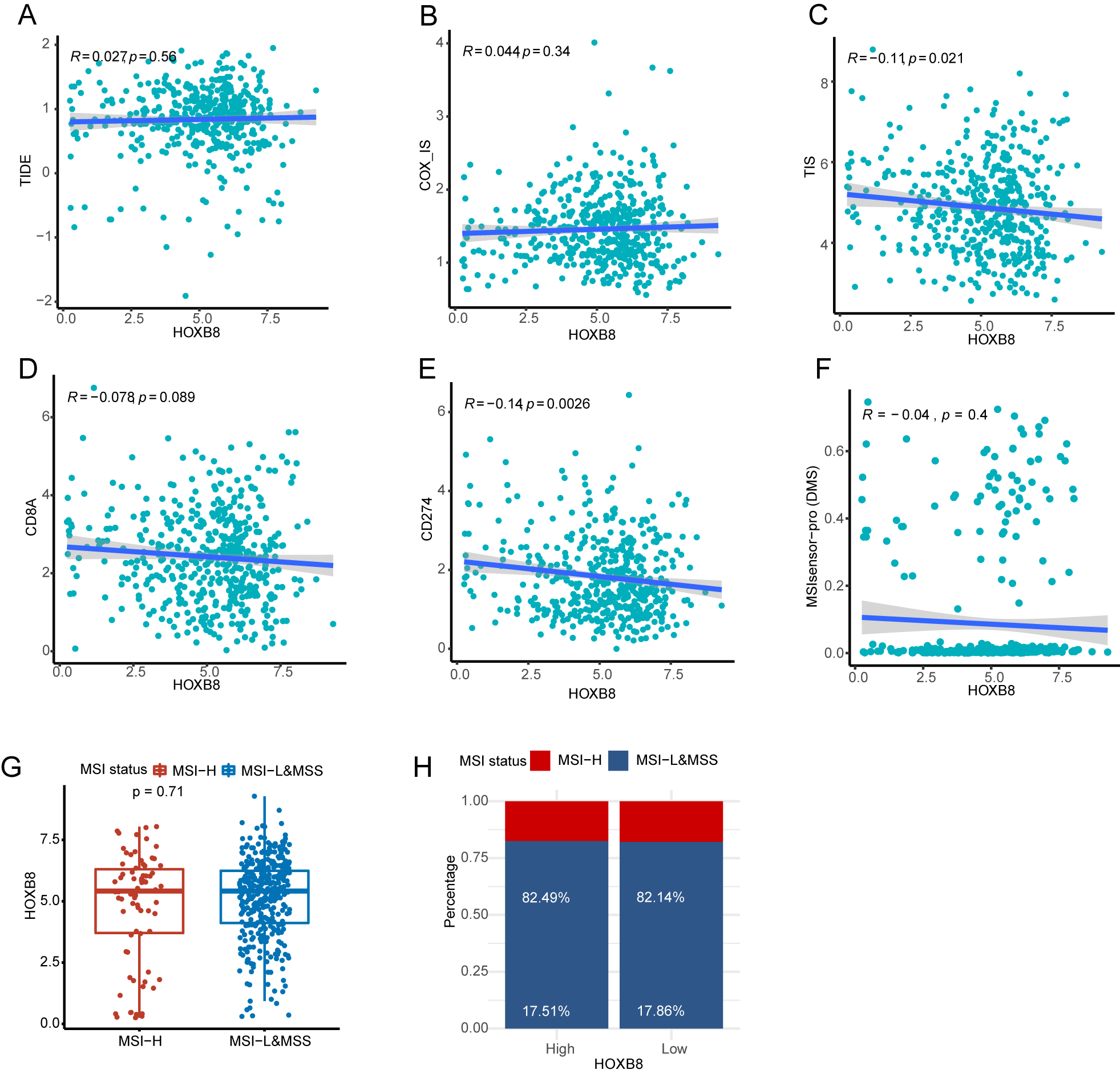

Supplement: Supplementary file 11 — Additional file 11. Fig. S11: Exploring the biological functions of HOXB8 in ERSGs scoring system. A–F The Pearson correlation between HOXB8 expression and TIDE score, COX-IS, TIS, CD8A, PD-L1 (CD274), and MSI score in TCGA COAD cohort. G The expression of HOXB8 between MSI-H and MSI-L/MSS patients. H The stacked histogram shows the distribution of MSI-H and MSI-L/MSS patients in the high and low expression of HOXB8 groups. [file 12967_2023_4547_MOESM11_ESM.tif]

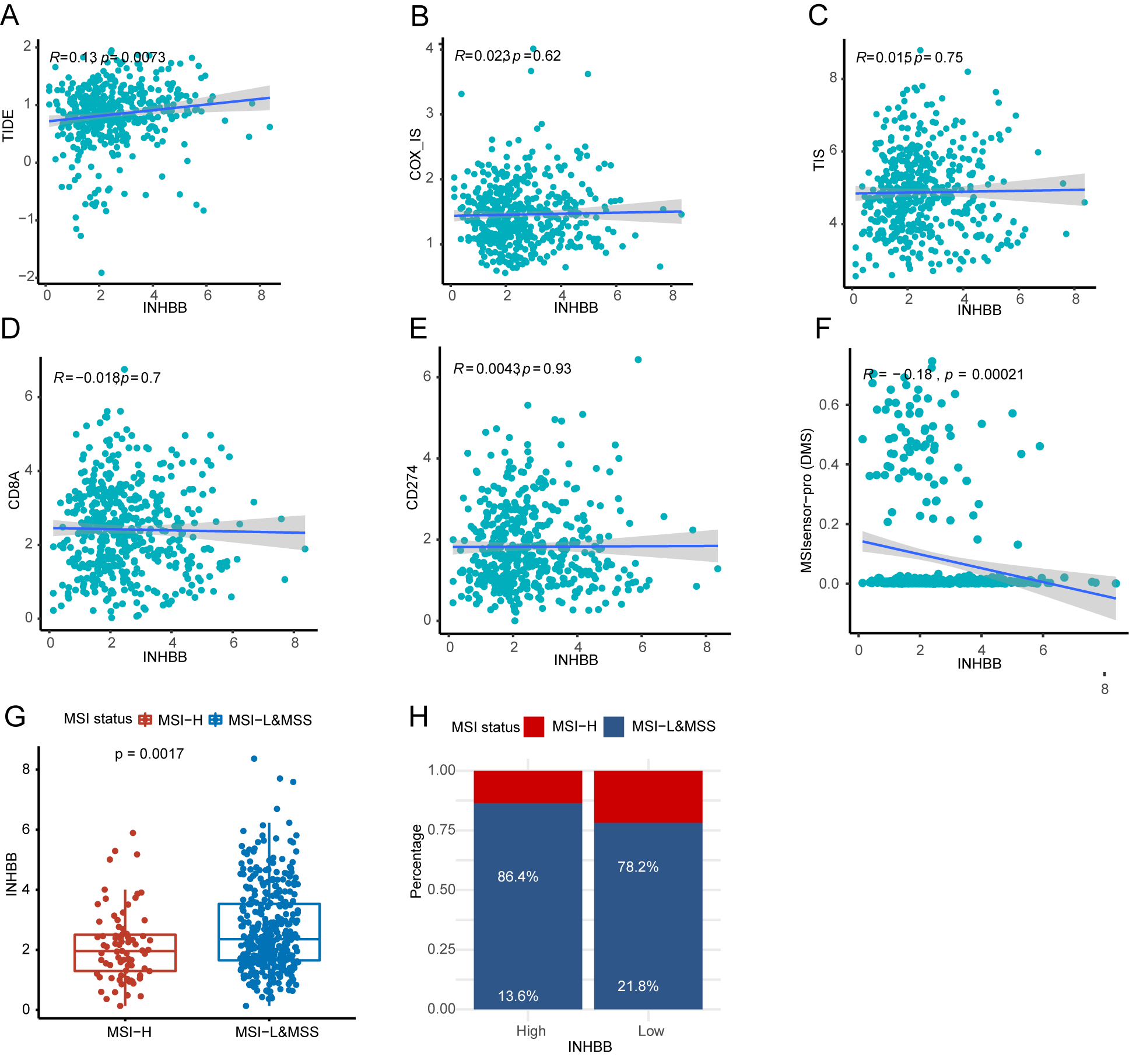

Supplement: Supplementary file 12 — Additional file 12. Fig. S12: Exploring the biological functions of INHBB in ERSGs scoring system. A–F The Pearson correlation between INHBB expression and TIDE score, COX-IS, TIS, CD8A, PD-L1 (CD274), and MSI score in TCGA COAD cohort. G The expression of INHBB between MSI-H and MSI-L/MSS patients. H The stacked histogram shows the distribution of MSI-H and MSI-L/MSS patients in the high and low expression of INHBB groups. [file 12967_2023_4547_MOESM12_ESM.tif]

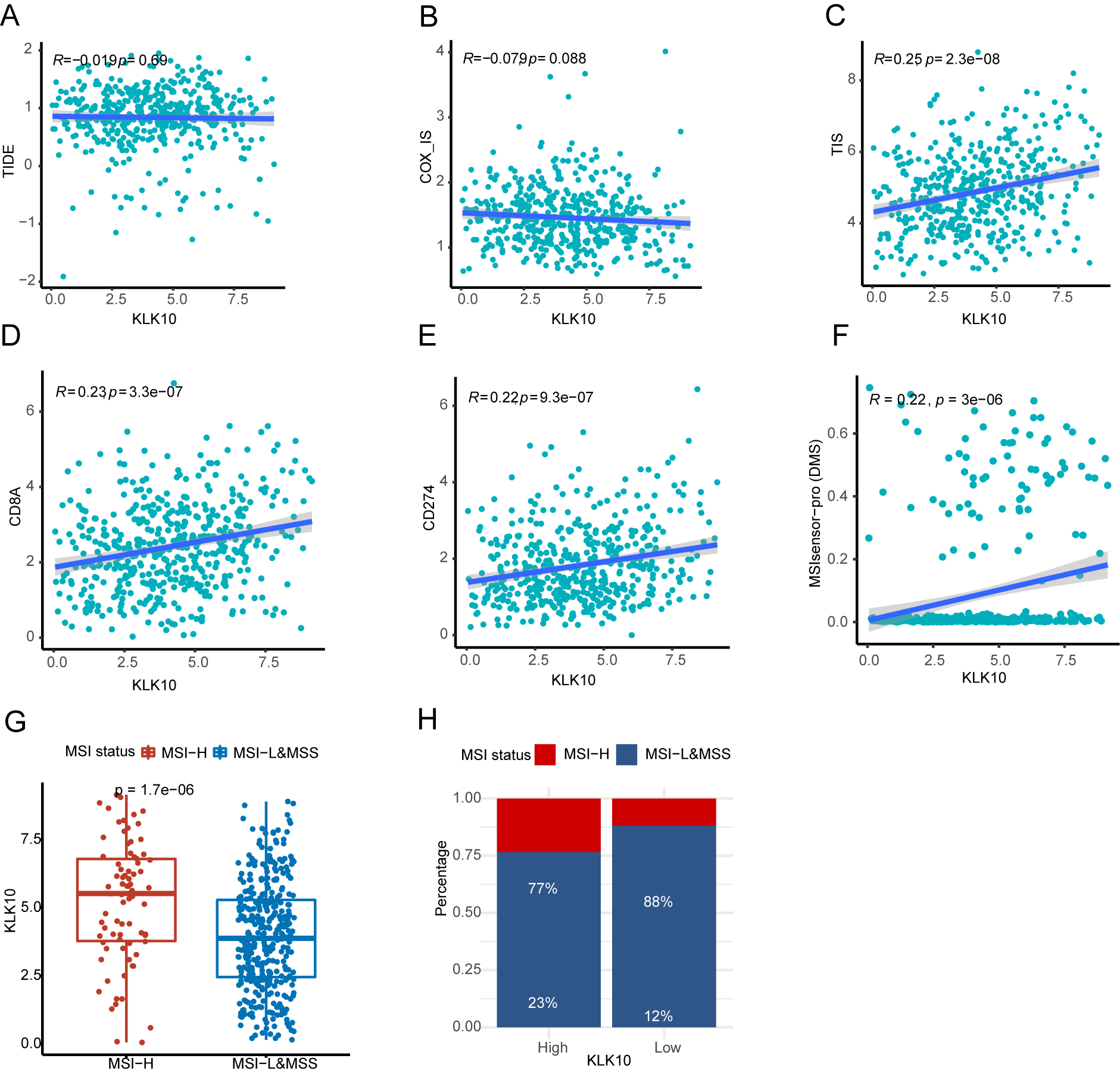

Supplement: Supplementary file 13 — Additional file 13. Fig. S13: Exploring the biological functions of KLK10 in ERSGs scoring system. A–F The Pearson correlation between KLK10 expression and TIDE score, COX-IS, TIS, CD8A, PD-L1 (CD274), and MSI score in TCGA COAD cohort. G The expression of KLK10 between MSI-H and MSI-L/MSS patients. H The stacked histogram shows the distribution of MSI-H and MSI-L/MSS patients in the high and low expression of KLK10 groups. [file 12967_2023_4547_MOESM13_ESM.tif]

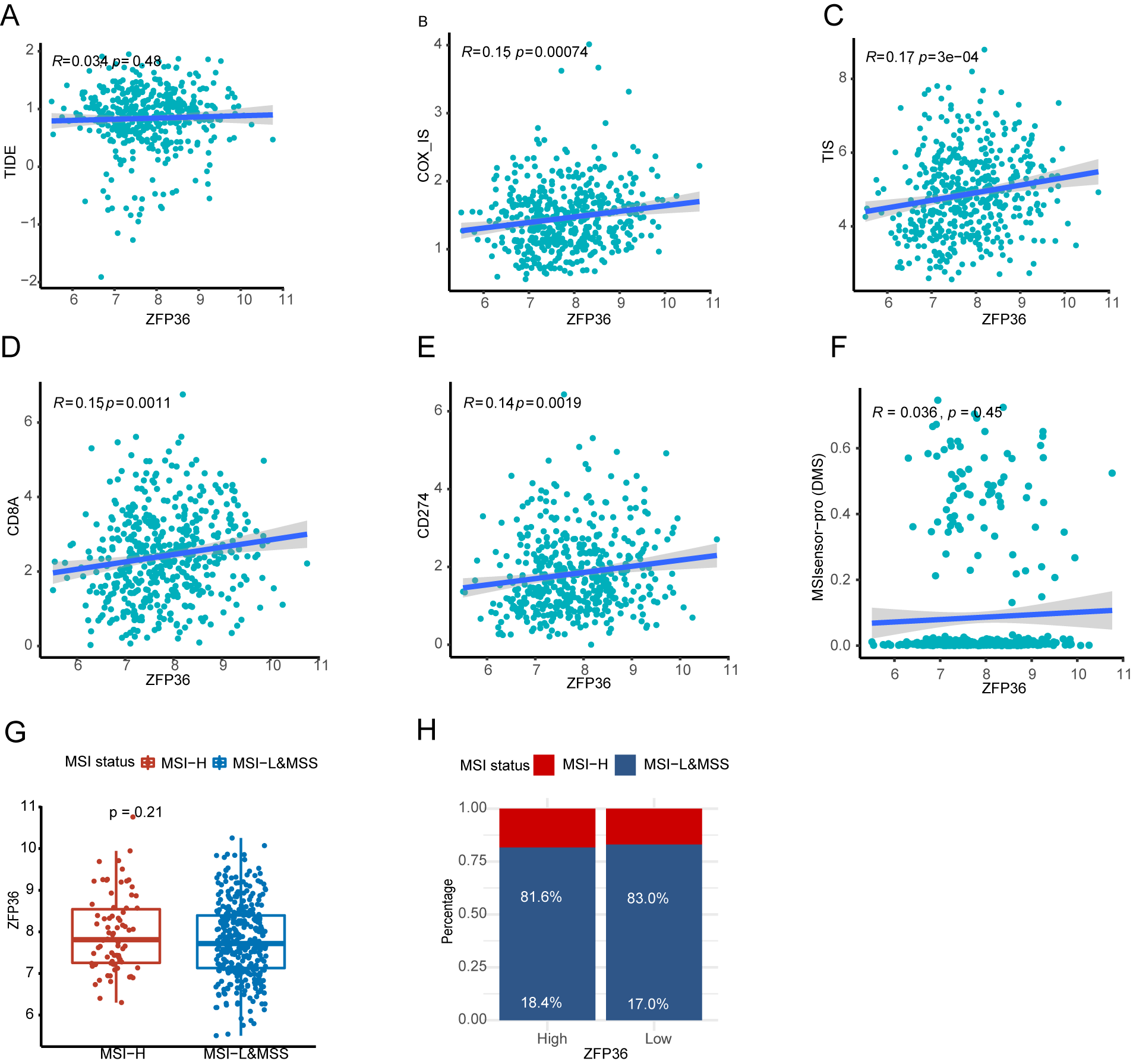

Supplement: Supplementary file 14 — Additional file 14. Fig. S14: Exploring the biological functions of ZFP36 in ERSGs scoring system. A–F The Pearson correlation between ZFP36 expression and TIDE score, COX-IS, TIS, CD8A, PD-L1 (CD274), and MSI score in TCGA COAD cohort. G The expression of ZFP36 between MSI-H and MSI-L/MSS patients. H The stacked histogram shows the distribution of MSI-H and MSI-L/MSS patients in the high and low expression of ZFP36 groups. [file 12967_2023_4547_MOESM14_ESM.tif]

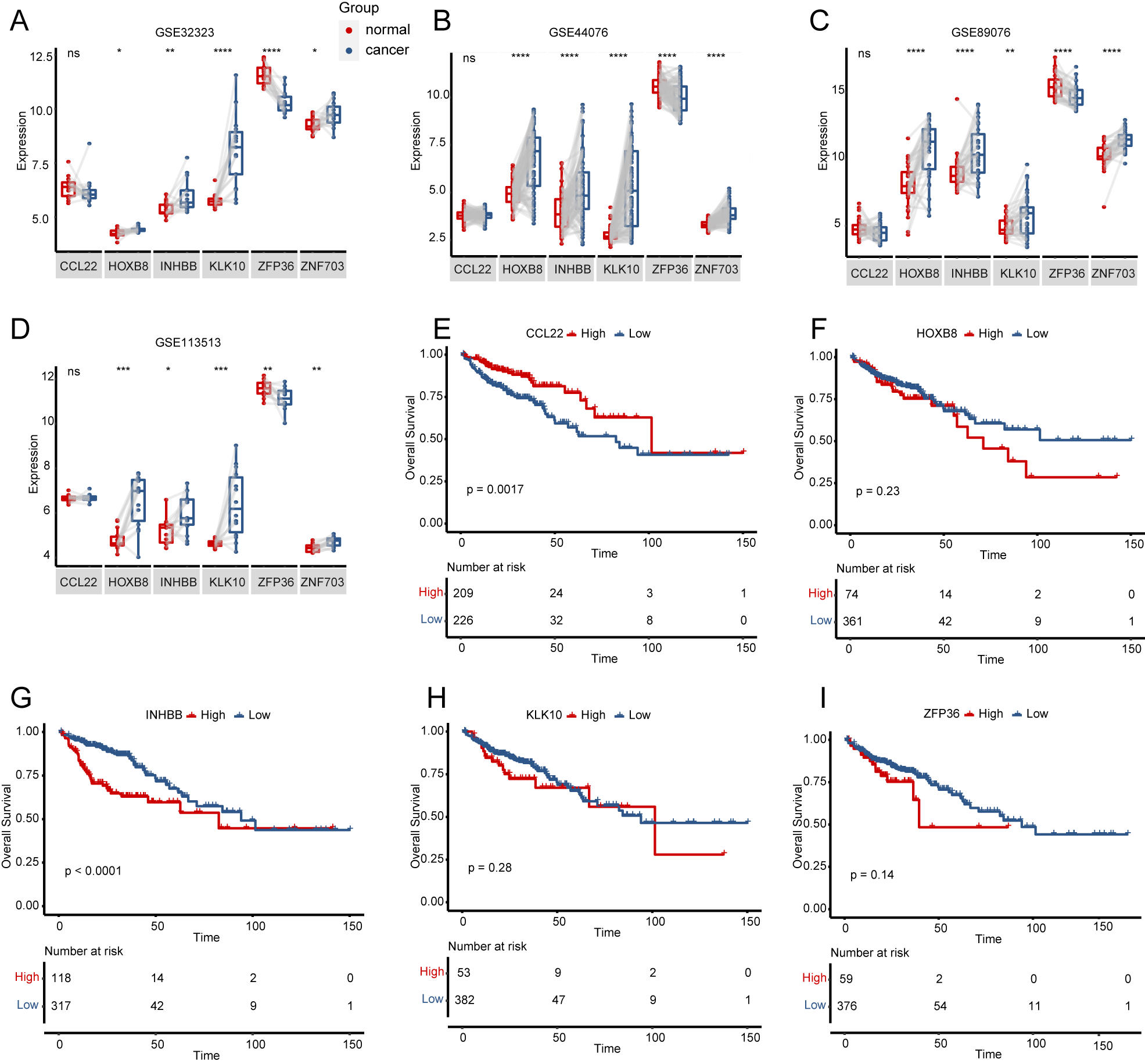

Supplement: Supplementary file 15 — Additional file 15. Fig. S15: Expression levels and survival analysis of biomarker genes in ERSGs scoring system. A–D The mRNA expressions of the six biomarker genes in ERSGs scoring system between normal and cancer tissue in GSE32323, GSE44076, GSE89076, and GSE113513 cohorts. E–I KM plots show the OS analysis of the high and low expression of CCL22, HOXB8, INHBB, KLK10, and ZFP36 groups in the TCGA COAD cohort. [file 12967_2023_4547_MOESM15_ESM.tif]
