# Supplementary material for: Characterization of endoplasmic reticulum stress unveils ZNF703 as a promising target for colorectal cancer immunotherapy
Source: J Transl Med. 2023 Oct 11;21:713. doi: 10.1186/s12967-023-04547-z (PMC10566095; doi:10.1186/s12967-023-04547-z)
Supplement: Supplementary file 16 — Additional file 16. Table S1: Detail information of public CRC cohorts used in this study. [file 12967_2023_4547_MOESM16_ESM.docx]

| CRC cohorts | Experiment type | Company/Platform | | Sample | Usage |
| --- | --- | --- | --- | --- | --- |
| GSE17536 | array | Affymetrix-GPL570 | 177 | | GEO combined cobort |
| GSE17537 | array | Affymetrix-GPL570 | 55 | | GEO combined cobort |
| GSE14333 | array | Affymetrix-GPL570 | 29 | | GEO combined cobort |
| GSE39582 | array | Affymetrix-GPL570 | 58 | | GEO combined cobort |
| GSE72968 | array | Affymetrix-GPL570 | 68 | | GEO combined cobort |
| TCGA-COAD | sequencing | Illumina | 571 | | Survival analysis  Differential expression analysis |
| GSE44076 | array | Affymetrix- GPL13667 | 246 | | Paired differential expression analysis (196 samples) |
| GSE32323 | array | Affymetrix- GPL570 | 44 | | Paired differential expression analysis (34 samples) |
| GSE89076 | array | Agilent- GPL16699 | 80 | | Paired differential expression analysis (74 samples) |
| GSE113513 | array | Affymetrix- GPL15207 | 28 | | Paired differential expression analysis (28 samples) |
